# Substrate displacement of CK1 C-termini regulates kinase specificity

**DOI:** 10.1126/sciadv.adj5185

**Published:** 2024-05-10

**Authors:** Sierra N. Cullati, Kazutoshi Akizuki, Jun-Song Chen, Jared L. Johnson, Tomer M. Yaron-Barir, Lewis C. Cantley, Kathleen L. Gould

**Affiliations:** ^1^ Department of Cell and Developmental Biology, Vanderbilt University School of Medicine, Nashville, TN, USA.; ^2^ Meyer Cancer Center, Weill Cornell Medicine, New York, NY, USA.; ^3^ Department of Cell Biology, Harvard Medical School, Boston, MA, USA.; ^4^ Dana-Farber Cancer Institute, Harvard Medical School, Boston, MA, USA.; ^5^ Englander Institute for Precision Medicine, Institute for Computational Biomedicine, Weill Cornell Medicine, New York, NY, USA.; ^6^ Columbia University Vagelos College of Physicians and Surgeons, New York, NY, USA.

## Abstract

CK1 kinases participate in many signaling pathways, and their regulation is of meaningful biological consequence. CK1s autophosphorylate their C-terminal noncatalytic tails, and eliminating these tails increases substrate phosphorylation in vitro, suggesting that the autophosphorylated C-termini act as inhibitory pseudosubstrates. To test this prediction, we comprehensively identified the autophosphorylation sites on *Schizosaccharomyces pombe* Hhp1 and human CK1ε. Phosphoablating mutations increased Hhp1 and CK1ε activity toward substrates. Peptides corresponding to the C-termini interacted with the kinase domains only when phosphorylated, and substrates competitively inhibited binding of the autophosphorylated tails to the substrate binding grooves. Tail autophosphorylation influenced the catalytic efficiency with which CK1s targeted different substrates, and truncating the tail of CK1δ broadened its linear peptide substrate motif, indicating that tails contribute to substrate specificity as well. Considering autophosphorylation of both T220 in the catalytic domain and C-terminal sites, we propose a displacement specificity model to describe how autophosphorylation modulates substrate specificity for the CK1 family.

## Teaser

This study proposes a model for CK1 substrate specificity based on autophosphorylation state.

## INTRODUCTION

CK1 enzymes are conserved, ubiquitous kinases that regulate essential cellular pathways including Wnt signaling, cell division, endocytosis, circadian rhythms, and DNA repair (*1*, *2*). Like other multifunctional kinases, CK1s must be regulated in space and time to specifically target their substrates in each of the pathways they participate in. Because CK1s prefer substrate motifs that have been previously phosphorylated (*3*), the action of substrate-priming kinases, or the opposing phosphatases, is considered a key mechanism of achieving CK1 substrate specificity (*4*–*7*). However, several important substrates [e.g., PER2 in circadian rhythms (*8*), LRP6 in Wnt signaling (*9*), Sid4 in a mitotic checkpoint (*10*), and Rec11 in meiotic recombination (*11*, *12*)] are not primed by other kinases, indicating that there must exist other mechanisms to control CK1 activity.

CK1 family members have related catalytic domains (53% to 98% sequence identity), a conserved extension to that kinase domain (KDE) that is important for enzyme stability and activity (*1*, *2*, *13*–*15*), and divergent C-terminal tails. In addition to catalytic domain autophosphorylation that occurs in many CK1 enzymes (*16*), all C-terminal tails appear to serve as substrates of autophosphorylation, and the phosphorylated C-termini are proposed to inhibit enzyme activity by acting as pseudosubstrates (*17*–*20*).

While there is abundant evidence that CK1 enzymes autophosphorylate their C-termini, and truncation or dephosphorylation of the tail increases kinase activity in vitro (*17*–*20*), an interaction between the phosphorylated tail and the kinase domain has not been demonstrated, and questions remain about how this regulatory mechanism functions. It is unclear whether truncation of the tail and dephosphorylation have equivalent effects on the enzyme. The affinity with which this interaction may occur in the context of substrate binding, and therefore how the plethora of CK1 substrates are regulated by autophosphorylation, is only beginning to be understood. While some autophosphorylation sites have been identified in CK1ε (*18*), the full complement of autophosphorylation sites, their cellular functions, and how autophosphorylation may affect different substrates has not been determined for any CK1 enzyme.

To investigate how CK1 family proteins are regulated by their C-termini, we focused on human CK1ε and *Schizosaccharomyces pombe* Hhp1 as representative CK1 enzymes. Hhp1 is one of two soluble CK1 enzymes in *S. pombe* (Hhp2 is the other), and these two yeast enzymes are highly related to CK1δ and CK1ε in human (*20*, *21*). Autophosphorylation of all four of these enzymes has been observed previously (*16*–*20*). Here, we identified all residues in the Hhp1 and CKε C-termini that are autophosphorylated in vitro; these sites are also targeted in vivo. Preventing phosphorylation of these specific sites increased kinase activity toward substrates. Reciprocally, the respective C-terminal phosphopeptides bound to their kinase domains, and this binding competitively inhibited substrate phosphorylation. We confirmed that autophosphorylated C-terminal peptides and substrates interacted with the kinase domain via the same positively charged amino acids in the binding groove. Critically, we found that substrates have a much greater affinity for the substrate binding groove than the phosphorylated C-termini, and that competition between the autophosphorylated C-termini and substrates for access to the kinase domains regulated the efficiency with which different substrates were targeted. This supports an updated paradigm for the regulation of CK1 family kinases via autophosphorylation, in which the phosphostate of CK1 can influence which substrates have high affinity for the catalytic domain.

## RESULTS

### Hhp1 autophosphorylates six sites on its C-terminus

**Fig. 1.**
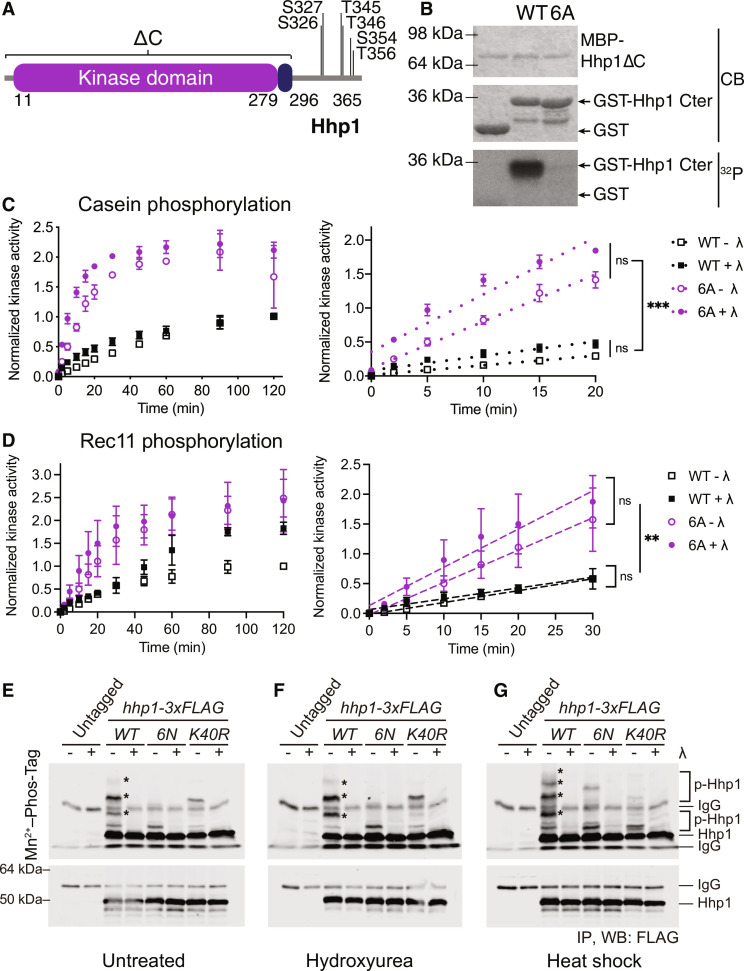
Autophosphorylation at six sites inhibits Hhp1 activity. (**A**) Domain structure of *S. pombe* Hhp1 showing autophosphorylation sites identified by mass spectrometry and confirmed by phosphopeptide mapping. (**B**) MBP-Hhp1ΔC was incubated with GST-Hhp1-Cter and [γ-^32^P]-ATP at 30°C for 2 hours. Phosphorylation was detected by autoradiography (^32^P) and total protein by Coomassie (CB). (**C** and **D**) MBP-Hhp1-WT or MBP-Hhp1-6A was treated ± λ phosphatase (λ) and then incubated with substrate and [γ-^32^P]-ATP at 30°C. Reactions were quenched at time points from 0 to 120 min, and casein phosphorylation (C) or Rec11 phosphorylation (D) was measured on a phosphorimager. Data from three independent replicates are shown as the mean ± SD. The full time course is shown on the left; the initial rate was determined by the slope of the linear section of the curve shown on the right. **P < 0.001, ***P < 0.0002, ns = not significant by one-way analysis of variance (ANOVA) of slopes. (**E** to **G**) *hhp1-wt*, *hhp1-6N*, and *hhp1-K40R* were tagged with 3xFLAG, and the resultant proteins were immunoprecipitated from denatured lysates with anti-FLAG antibody. IPs were treated ± λ, and electrophoretic mobility shifts on 6% gels containing 40 μM Phos-Tag were detected with anti-FLAG antibody (top). Total Hhp1-3xFLAG was visualized on gels without Phos-Tag (bottom). Asterisk (*) indicates autophosphorylated Hhp1-3xFLAG. (F) Cells were treated with 12 mM HU for 3.5 hours before IP. (G) Cells were incubated at 44°C for 30 min before IP.

CK1 enzymes phosphorylate their C-terminal, noncatalytic tails (*17*–*20*), but the full complement of autophosphorylation sites has not been identified for any one enzyme. To identify autophosphorylation sites on *S. pombe* Hhp1, we analyzed the recombinant kinase by mass spectrometry (fig. S1A). Many of the phosphopeptides that we identified contained multiple serines and threonines, leading to ambiguous localization of phosphorylation sites. Therefore, we eliminated false positives and verified candidate sites by generating alanine substitution mutants at each candidate site and performing phosphopeptide mapping of the autophosphorylated, recombinant proteins (fig. S1B). In total, we found that six sites in the Hhp1 C-terminus can be autophosphorylated: Ser^326^, Ser^327^, Thr^345^, Thr^346^, Ser^354^, and Thr^356^ (Fig. 1A). Phosphorylated Ser^354^ and Thr^356^ have also been observed in large-scale phosphoproteomics experiments (*22*–*25*). A mutant with all six sites mutated to alanine (Hhp1-6A) produces an autophosphorylation map identical to that of C-terminally truncated Hhp1 (Hhp1ΔC, amino acids 1 to 296) (fig. S1B). The remaining phosphopeptides are due to Thr^221^ and Thr^222^ autophosphorylation of the kinase domain (*16*). Not only could CK1 autophosphorylate the C-terminal sites in the full-length protein, but also we expressed and purified amino acids 297 to 365 of Hhp1 (Cter) fused to glutathione S-transferase (GST), and Hhp1ΔC was able to use wild-type Cter, but not Cter-6A, as a substrate in trans (Fig. 1B). These data support the conclusion that all Hhp1 C-terminal sites that can be autophosphorylated in vitro were identified.

Truncation or phosphatase treatment of CK1δ, CK1ε, Hhp1, and Hhp2 increases phosphorylation of the model substrate casein (*16*–*20*). This predicts that preventing autophosphorylation of the Hhp1 C-terminal sites would similarly increase Hhp1’s ability to phosphorylate casein and other substrates. We measured the rate of casein phosphorylation by recombinant Hhp1-6A and Hhp1-6N, obtaining identical results with these two mutants in vitro (Fig. 1C and fig. S2A). Consistent with the literature, Hhp1-6A and Hhp1-6N were about twofold more active than the wild-type enzyme, and the initial velocities of substrate phosphorylation by Hhp1-6A and Hhp1-6N were significantly greater than that of Hhp1-WT (Fig. 1C). These results were recapitulated using the N terminus of Rec11 (GST-Rec11-N3, consisting of amino acids 1–33) (*12*), a physiological Hhp1 substrate involved in meiosis (Fig. 1D and fig. S2B) (*11*, *12*). Even after initial dephosphorylation with λ phosphatase, Hhp1-WT rapidly rephosphorylates C-terminal sites (fig. S2C) and catalytic domain sites (*16*) during the course of the experiment (*4*, *17*, *20*, *26*, *27*). Because Hhp1-6A and Hhp1-6N cannot, these mutants were even more active than dephosphorylated wild-type Hhp1 (Fig. 1, C and D and fig. S2, A and B).

On the basis of these in vitro data, we predicted that *hhp1-6A* and *hhp1-6N* would be gain-of-function alleles. When we replaced the endogenous *hhp1* gene with *hhp1-6A* in haploid *S. pombe* cells and created heterozygous and homozygous diploid strains, we found that this allele conferred sensitivity to hydroxyurea (HU), consistent with a recessive loss of function (fig. S3, A and B). In contrast, *hhp1-6N* grew more slowly than wild type at all temperatures but was not sensitive to HU (fig. S3C). *hhp2* is the paralog of *hhp1* that has redundant functions; deleting it in *hhp1-6N* cells rescued the slow growth of *hhp1-6N*, consistent with the idea that Hhp1-6N exhibits increased activity in vivo that is detrimental to cells. Furthermore, the *hhp1-6N* allele allowed *hhp2*Δ to grow better on plates containing HU, and even high levels of HU did not slow the growth of *hhp1-6N*, demonstrating that it is not a loss-of-function allele (fig. S3C). We surmise that the more polar amino acid asparagine preserves function better than alanine; therefore, we used *hhp1-6N* for further in vivo studies.

To determine whether the C-terminal autophosphorylation sites identified in vitro were also targeted in vivo, we appended sequences encoding 3xFLAG to the *hhp1, hhp1-6N,* and kinase-dead *hhp1-K40R* alleles at their endogenous loci and examined the electrophoretic mobility of the proteins. Immunoprecipitated Hhp1 WT-3xFLAG exhibited several slowly migrating bands on a gel containing Phos-Tag reagent (Fig. 1E). Treating with λ phosphatase collapsed these bands, confirming that they are the result of phosphorylation. The migration pattern of Hhp1-6N-3xFLAG was very different, having only one band with slightly reduced mobility, indicating that the sites we identified are phosphorylated in vivo (Fig. 1E). To test if they were phosphorylated by Hhp1 itself, we compared the migration of Hhp1-K40R-3xFLAG and found that it was very similar to Hhp1-6N-3xFLAG (Fig. 1E). Both Hhp1-6N-3xFLAG and Hhp1-K40R-3xFLAG lost the major phosphorylated species present in Hhp1 WT-3xFLAG while retaining a minor phosphorylated species, suggesting that Hhp1 can also be phosphorylated by other cellular kinases. The basal level of phosphorylated Hhp1 was small; however, when we treated cells with HU, which induces replication stress, or with heat stress, the proportion of phosphorylated Hhp1-3xFLAG species increased (Fig. 1, F and G). The amount of highly phosphorylated Hhp1 during heat stress increased with time (fig. S3D), and the constitutively dephosphorylated *hhp1-6N* strain was heat resistant (fig. S3E), which we also observed for *hhp1* alleles that lack kinase domain autophosphorylation (*16*). This demonstrates that autophosphorylation of the Hhp1 C-terminus on these six sites is dynamic in cells and can regulate cellular processes such as the response to heat shock.

### The C-terminus of Hhp1 interacts with the kinase domain in a phosphorylation-dependent manner

**Fig. 2.**
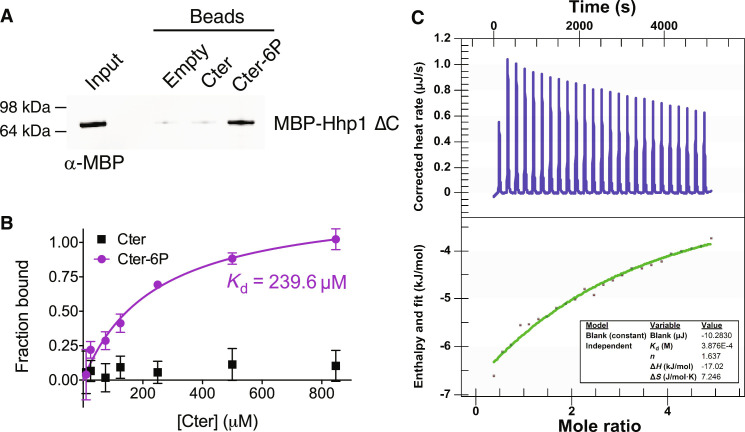
The Hhp1 C-terminus interacts with the kinase domain in a phosphorylation-dependent manner. (**A**) Hhp1 Cter or Cter-6P was conjugated to beads and incubated with MBP-Hhp1ΔC at 4°C for 1 hour. Bound protein was visualized by Western blot. Representative blot from three independent replicates. (**B**) Various concentrations of Hhp1 Cter or Cter-6P beads were incubated with MBP-Hhp1ΔC at 4°C for 1 hour. Protein remaining in the supernatant was visualized by Western blot and quantified by densitometry. Data from three independent replicates (mean ± SD) was fitted to a one-site specific binding curve to calculate the *K*_d_. (**C**) Representative ITC experiment with MBP-Hhp1ΔC and Cter-6P. Top panel shows raw data; bottom panel shows normalized integrated data. See fig. S4 (A and B) for additional replicate and negative controls.

The idea that autophosphorylated CK1 C-termini can act as pseudosubstrates predicts that they interact with their kinase domains (*17*–*20*). To test this prediction for Hhp1, we used synthetic, biotin-conjugated peptides corresponding to the unphosphorylated Hhp1 Cter and the fully phosphorylated Hhp1 Cter (Cter-6P). When linked to streptavidin beads, Hhp1 Cter-6P, but not unphosphorylated Hhp1 Cter, pulled down Hhp1ΔC from solution (Fig. 2A). This result supports a direct, autophosphorylation-dependent interaction between the C-terminus and the kinase domain. We performed equilibrium binding assays at multiple concentrations of Cter to measure the dissociation constant (*K*_d_) of the Cter–kinase domain interaction. While the unphosphorylated Hhp1 Cter showed no appreciable binding even at the highest concentration, Hhp1 Cter-6P binding fit a one-site specific binding curve with *K*_d_ = 240 μM (Fig. 2B). To estimate the binding affinity with both partners in solution, we performed isothermal titration calorimetry (ITC), and the *K*_d_ for Hhp1 Cter-6P determined via this method, 335 ± 74 μM, was consistent with the *K*_d_ determined via pull-down (Fig. 2C and fig. S4A). Because of the high micromolar affinity of this interaction, the stoichiometry values obtained by ITC were likely not reliable, and there was also some variance in the calculated *K*_d_. However, we detected no binding of the unphosphorylated Cter (fig. S4B), which was clearly different than Cter-6P, so we conclude that there is a low affinity interaction between the autophosphorylated C-terminus and the kinase domain, on the order of hundreds of micromolar.

### The C-terminus of Hhp1 competes for binding to the substrate binding groove

**Fig. 3.**
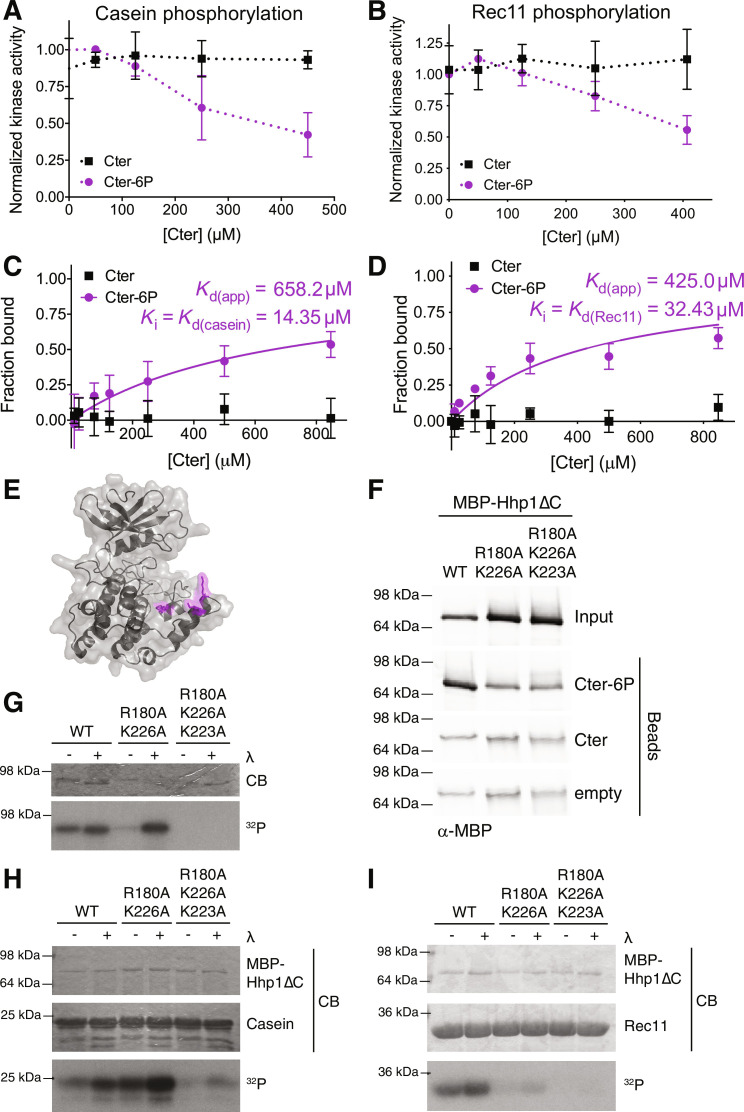
The Hhp1 C-terminus competes with substrates to bind the substrate binding groove. (**A** and **B**) Various concentrations of soluble Hhp1 Cter or Cter-6P were incubated with MBP-Hhp1ΔC, casein (A) or Rec11 (B), and [γ-^32^P]-ATP at 30°C for 1 hour. Substrate phosphorylation was measured on a phosphorimager. Data from three independent replicates are shown as the mean ± SD. (**C** and **D**) Various concentrations of Hhp1 Cter or Cter-6P beads were incubated with MBP-Hhp1ΔC in the presence of 25 μM casein (C) or Rec11 (D) at 4°C for 1 hour. Protein remaining in the supernatant was visualized by Western blot and quantified by densitometry. Data from three independent replicates (mean ± SD) were fitted to a one-site specific binding curve to calculate the apparent *K*_d_ for the kinase domain–Cter interaction, and the substrate *K*_i_ was then calculated based on the change in *K*_d_ compared to that measured in Fig. 2B. (**E**) Phyre2 homology model (*57*) of Hhp1, with the basic residues of the substrate binding groove highlighted in magenta. (**F**) Hhp1 Cter or Cter-6P was conjugated to beads and incubated with MBP-Hhp1ΔC with basic residues in the substrate binding groove mutated to alanine. Bound protein was visualized by Western blot. (**G**) MBP-Hhp1 was treated ± λ phosphatase (λ) and then incubated with [γ-^32^P]-ATP at 30°C for 45 min. Autophosphorylation was detected by autoradiography (^32^P) and total protein by Coomassie (CB). (**H** and **I**) MBP-Hhp1ΔC was treated ± λ and then incubated with casein (H) or Rec11 (I) and [γ-^32^P]-ATP at 30°C for 45 min. Substrate phosphorylation was detected by autoradiography (^32^P) and total protein by Coomassie (CB). For (F) to (I), representative data from three independent replicates are shown.

To test whether the interaction between the phosphorylated Cter and the kinase domain inhibited substrate phosphorylation, we incubated Hhp1ΔC with increasing concentrations of the soluble Hhp1 Cter peptides and either the model substrate casein or the physiological substrate Rec11. Hhp1 Cter-6P inhibited substrate phosphorylation in a dose-dependent manner, where casein and Rec11 phosphorylation decreased by approximately half at the *K*_d_ of the binding interaction (Fig. 3, A and B). Similar to the binding data (Fig. 2, A to C), unphosphorylated Hhp1 Cter did not inhibit substrate phosphorylation.

The pseudosubstrate model and structural studies predict that binding of the C-terminus to the CK1 kinase domain prevents substrate phosphorylation by directly competing with substrate for binding to the kinase domain (*27*–*29*). To confirm that this is the case in Hhp1, we repeated the equilibrium binding experiment in the presence of substrate (Fig. 3, C and D). Here, casein and Rec11 act as inhibitors to Cter binding, and we expected the apparent *K*_d_ for the Cter to increase due to competitive inhibition. We found that the *K*_d(app)_ for Cter-6P in the presence of 25 μM casein was 658 μM, and the *K*_i(casein)_, which is equal to the *K*_d(casein)_, was 14 μM (Fig. 3C). Similarly, *K*_i(Rec11)_ = *K*_d(Rec11)_ = 32 μM (Fig. 3D). It is noteworthy that the affinities of Hhp1ΔC for these substrates are an order of magnitude greater than for the autophosphorylated C-terminus.

Because the substrate binding groove is positively charged, mutating basic residues in this region (Arg^180^, Lys^223^, and Lys^226^ in Hhp1) to alanine should prevent both Cter-6P binding and substrate phosphorylation (Fig. 3E). To test this, we expressed and purified recombinant Hhp1ΔC-R180A, K223A, K226A. To confirm that the overall secondary structure of the mutant kinase remained intact, we performed circular dichroism at far-ultraviolet (UV) wavelengths. The spectra of Hhp1ΔC-R180A, K223A, K226A resembled that of wild-type Hhp1ΔC, indicating that the mutations had not disrupted the overall folding of the catalytic domain (fig. S5A). However, these mutations eliminated binding to the Cter-6P peptide compared to wild-type Hhp1ΔC (Fig. 3F) and prevented C-terminal autophosphorylation in full-length Hhp1 (Fig. 3G). Hhp1ΔC-R180A, K223A, K226A dramatically reduced phosphorylation of casein (Fig. 3H) and Rec11 (Fig. 3I). A mutant that partially disrupted the substrate binding groove (Hhp1ΔC-R180A, K226A) had the intriguing effect of increasing casein phosphorylation but decreasing Rec11 phosphorylation (Fig. 3, H to I). We hypothesize that this mutant disrupted product inhibition that limited the rate of casein phosphorylation, as has been demonstrated for other CK1 substrates (*28*–*30*), but did not fully prevent interaction of casein with Hhp1. These two mutations were, however, sufficient to fully disrupt the interaction of Rec11 and the C-terminus with the substrate binding groove, resulting in decreased phosphorylation of these substrates. Overall, these data are consistent with the proposal that the autophosphorylated C-termini of CK1s interact with the substrate binding grooves of the catalytic domains.

### Autophosphorylation-dependent, substrate-competitive tail binding is conserved in human CK1ε

**Fig. 4.**
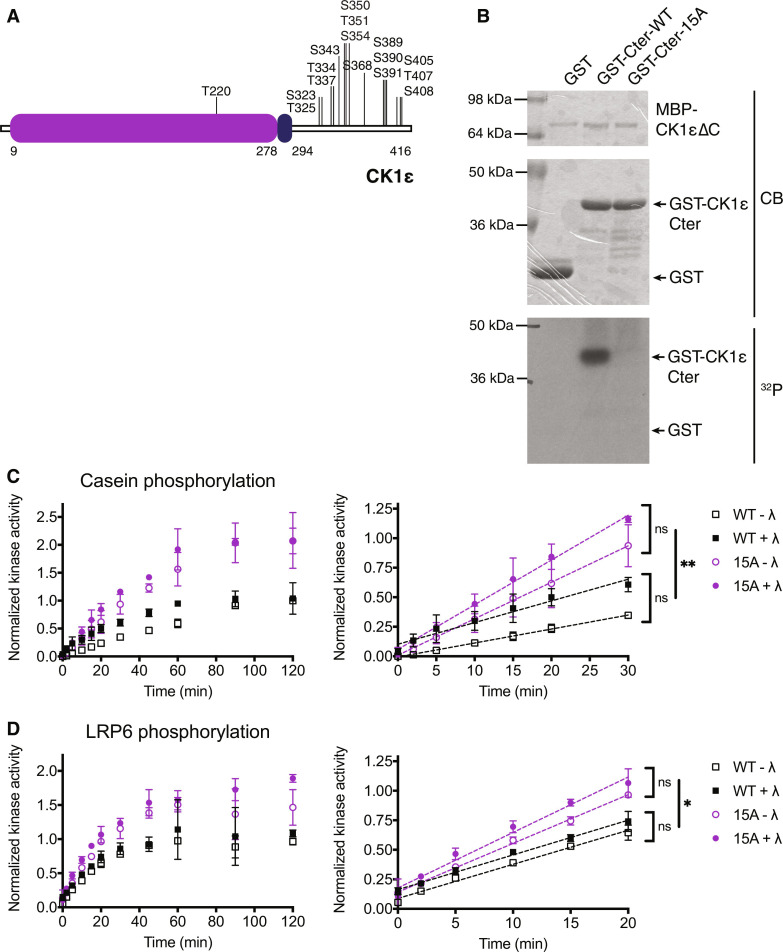
Autophosphorylation at 15 sites inhibits CK1ε activity. (**A**) Domain structure of human CK1ε showing confirmed autophosphorylation sites. (**B**) MBP-CK1εΔC was incubated with GST-CK1ε-Cter and [γ-^32^P]-ATP at 30°C for 2 hours. Phosphorylation was detected by autoradiography (^32^P) and total protein by Coomassie (CB). (**C** and **D**) MBP-CK1ε-WT or MBP-CK1ε-15A was treated ± λ phosphatase (λ) and then incubated with substrate and [γ-^32^P]-ATP at 30°C. Reactions were quenched at time points from 0 to 120 min, and casein phosphorylation (C) or LRP6 phosphorylation (D) was measured on a phosphorimager. Data from three independent replicates are shown as the mean ± SD. The full time course is on the left; the initial rate was determined by the slope of the linear section of the curve on the right. **P < 0.001, *P < 0.01, ns = not significant by one-way ANOVA of slopes.

To ask whether the autoinhibition model confirmed for Hhp1 was conserved in its human orthologs, we first needed to identify the C-terminal autophosphorylation sites in a human CK1. A previous study characterized CK1ε-MM2, which consisted of eight autophosphorylation sites mutated to alanine (Ser^323^, Thr^325^, Thr^334^, Thr^337^, Ser^368^, Ser^405^, Thr^407^, and Ser^408^) (*18*). We verified that these sites were autophosphorylated in vitro using phosphopeptide mapping; however, there were additional sites because the CK1ε-MM2 map was not identical to CK1εΔC (fig. S6A). Furthermore, CK1ε was found to incorporate up to 12 moles of phosphate per mole of enzyme (*17*), supporting the existence of additional phosphorylation sites. We immunoprecipitated CK1ε from human embryonic kidney (HEK) 293 cells and identified additional candidate phosphorylation sites by mass spectrometry. As for Hhp1, the tail of CK1ε produces long phosphopeptides containing multiple serines and threonines, making phosphorylation site localization challenging. We expressed alanine substitution mutants at each candidate site and performed phosphopeptide mapping of the autophosphorylated, recombinant proteins to verify that sites were the result of autophosphorylation and to accurately localize sites (fig. S6A). Ultimately, we mutated an additional seven sites (Ser^343^, Ser^350^, Thr^351^, Ser^354^, Ser^389^, Ser^390^, and Ser^391^) in combination with the eight sites in CK1ε-MM2 to generate CK1ε-15A (Fig. 4A), which abolished all C-terminal autophosphorylation (fig. S6A). Similar to Hhp1, CK1εΔC can phosphorylate its C-terminus in trans, but not when these 15 sites are mutated to alanine (Fig. 4B).

CK1ε-15A phosphorylated two substrates, casein (Fig. 4C) and the cytoplasmic domain of LRP6 (Fig. 4D), a CK1ε substrate involved in Wnt signaling (*5*, *9*, *31*, *32*), at a greater rate than did wild-type CK1ε, suggesting that autophosphorylation of the C-terminus at these 15 sites inhibits substrate phosphorylation.

**Fig. 5.**
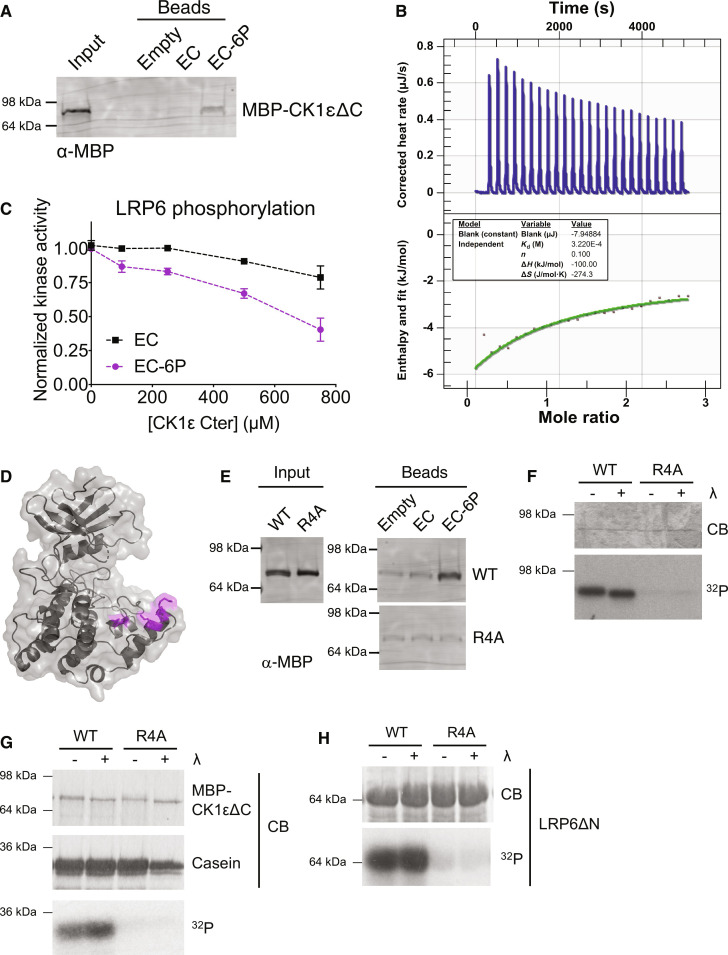
Phosphorylation-dependent tail binding and competition with substrate is conserved in human CK1ε. (**A**) CK1ε EC or EC-6P was conjugated to beads and incubated with MBP-CK1εΔC at 4°C for 1 hour. Bound protein was visualized by Western blot. Representative blot from two independent replicates. (**B**) Representative ITC experiment with MBP-CK1εΔC and EC-6P. Top panel shows raw data; bottom panel shows normalized integrated data. See fig. S4 (C and D) for additional replicate and negative controls. (**C**) Various concentrations of soluble CK1ε EC or EC-6P were incubated with MBP-CK1εΔC, LRP6, and [γ-^32^P]-ATP at 30°C for 1 hour. LRP6 phosphorylation was measured on a phosphorimager. Data from two independent replicates are shown as the mean ± SD. (**D**) Structural model of CK1ε (Protein Data Bank ID 4HOK) (*58*) with the basic residues of the substrate binding groove highlighted in magenta. (**E**) CK1ε EC or EC-6P was conjugated to beads and incubated with MBP-CK1εΔC with basic residues in the substrate binding groove mutated to alanine (R4A). Bound protein was visualized by Western blot. (**F**) MBP-CK1ε was treated ± λ phosphatase (λ) and then incubated with [γ-^32^P]-ATP at 30°C for 45 min. Autophosphorylation was detected by autoradiography (^32^P) and total protein by Coomassie (CB). (**G** and **H**) MBP-CK1εΔC was treated ± λ and then incubated with casein (G) or LRP6 (H) and [γ-^32^P]-ATP at 30°C for 45 min. Substrate phosphorylation was detected by autoradiography (^32^P) and total protein by Coomassie (CB). For (E) to (H), representative data from two independent replicates are shown.

We next tested the binding of CK1εΔC to the autophosphorylated C-terminus. Because of the length (122 amino acids) and number of phosphorylation sites, it was not possible to synthesize the entire autophosphorylated CK1ε Cter as we had for Hhp1 Cter. Instead, we used a peptide corresponding to the final 34 amino acids of CK1ε (called EC) due to the demonstrated effect of the extreme C-terminus on the phosphorylation of PER2 (*8*, *33*). The EC-6P peptide included the six autophosphorylation sites that occur in that region. Recombinant CK1εΔC interacted with EC-6P but not EC in a pulldown experiment (Fig. 5A), and using ITC, we estimated that the *K*_d_ of this interaction was 343 ± 30 μM (Fig. 5B and fig. S4, C and D). Increasing concentrations of EC-6P inhibited LRP6 phosphorylation by CK1εΔC (Fig. 5C).

Mutating basic residues in the substrate binding groove generated CK1εΔC-R178A, K221A, R222A, K224A (Fig. 5D), which was unable to interact with EC-6P (Fig. 5E) or autophosphorylate its C-terminus (Fig. 5F). This mutant also had dramatically reduced activity toward casein (Fig. 5G) and LRP6 (Fig. 5H) compared to wild type, although the overall folding of the mutant was similar to that of the wild type (fig. S5B). We conclude that as for Hhp1, the autophosphorylated C-terminus of CK1ε competes with substrates to bind the catalytic domain.

### CK1 C-termini contribute to substrate specificity

**Fig. 6.**
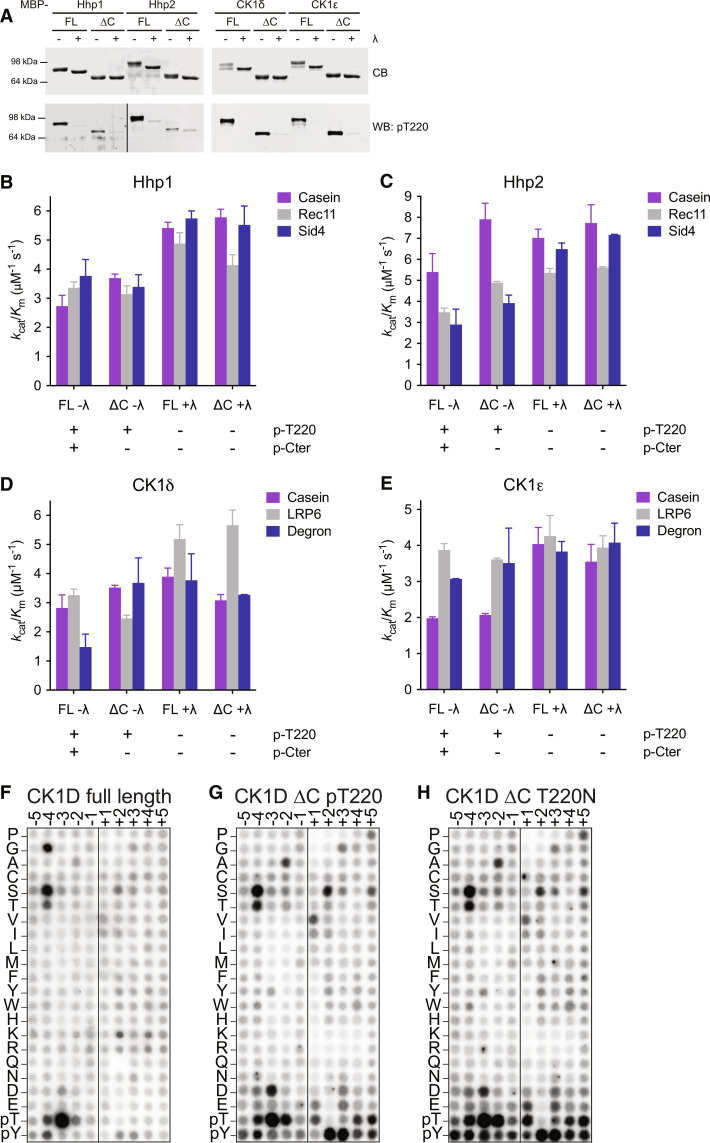
CK1 C-termini contribute to substrate specificity. (**A**) To confirm the phosphorylation status of each enzyme, the indicated CK1s were treated ± λ phosphatase (λ) for 45 min at 30°C and then quenched by adding phosphatase inhibitor and SDS-PAGE sample buffer. Proteins were separated by SDS-PAGE, followed by Coomassie staining (CB) to visualize phosphorylation-dependent electrophoretic mobility shifts and Western blot (WB) using anti-pT220 antibody. (**B** to **E**) Specificity footprints for Hhp1 (B), Hhp2 (C), CK1δ (D), and CK1ε (E). The indicated CK1 enzymes were treated ± λ as in (A) and then incubated with the indicated substrates and [γ-^32^P]-ATP at 30°C. Reactions were quenched at time points from 0 to 60 min, and substrate phosphorylation was measured on a phosphorimager. The initial rate was determined by the slope of the linear section of the curve and then used to calculate the *k*_cat_/*K*_m_ (see Materials and Methods for details). Data from three independent replicates are shown as the mean ± SD. (**F** to **H**) Peptide substrate specificities for CK1δ (F), CK1δΔC pT220 (G), and CK1δΔC-T220N (H). Amino acids are shown on the *y* axis, while position relative to the phosphorylated serine (vertical line) is shown on the *x* axis. Darker spots indicate preferred residues.

The competitive binding interactions between the C-termini of Hhp1 and CK1ε and their substrates suggested that the C-termini might influence which substrates were targeted by each kinase. In other words, are all substrates equally competed by the phosphorylated C-termini, or are some more easily prevented from accessing the substrate binding groove than others? To answer this question, we measured the catalytic efficiency or specificity constant (*k*_cat_/*K*_m_) for the full-length and ΔC versions of Hhp1, Hhp2, CK1δ, and CK1ε on a few representative substrates. In addition, we treated with λ phosphatase to remove T220 autophosphorylation, which influences substrate specificity by changing the conformation of the substrate binding groove (*16*). We confirmed the autophosphorylation status of each condition using a pT220 phospho-specific antibody (*16*) and by observing the electrophoretic mobility shift that depends on C-terminal autophosphorylation (Fig. 6A).

We directly measured *k*_cat_/*K*_m_ by using a very low substrate concentration, which also allowed utilization of additional substrates that were less biochemically tractable to build a “specificity footprint” for each phospho-form of each kinase (Fig. 6, B to E). To reduce complexity, the substrates that we chose did not require priming by other kinases. In addition to casein and Rec11, we used Sid4 for Hhp1 and Hhp2, which is a substrate involved in a yeast mitotic checkpoint (*10*). For CK1δ and CK1ε, we used casein, LRP6, and a peptide from PER2 that includes the β-TrCP phosphodegron site (*29*).

We tested whether truncating the C-termini affected the catalytic efficiency in the same way as abolishing the C-terminal autophosphorylation sites. We measured similar *k*_cat_/*K*_m_ values for Hhp1-6A and Hhp1ΔC (fig. S7A and Fig. 6B) as well as for CK1ε-15A and CK1εΔC (fig. S7B and Fig. 6E). This allowed us to compare the *k*_cat_/*K*_m_ for the different phospho-forms of Hhp2 and CKδ, even without knowing their C-terminal autophosphorylation sites. We also measured the *k*_cat_/*K*_m_ for casein using the Hhp1 pCter peptide with Hhp1ΔC in trans and compared it to full-length Hhp1. The pCter peptide suppressed the *k*_cat_/*K*_m_ to a similar extent as in autophosphorylated full-length Hhp1 (fig. S7C), suggesting that the binding data using the pCter peptide reasonably represent the physiological situation, in which the C-terminus is autophosphorylated and bound in cis.

We found that the catalytic efficiencies were different for different substrates—for example, full-length autophosphorylated Hhp1 was more likely to phosphorylate Sid4 than casein (Fig. 6B). Removing either C-terminal autophosphorylation or T220 autophosphorylation tended to increase the *k*_cat_/*K*_m_ for all substrates, but the magnitude of this effect differed. For example, Hhp1ΔC had a ~30% greater *k*_cat_/*K*_m_ for casein than Hhp1, but the efficiency of Rec11 and Sid4 phosphorylation was nearly the same for Hhp1ΔC and Hhp1 such that Hhp1ΔC had little preference for one substrate over the other (Fig. 6B). Dephosphorylating the T220 sites further increased the *k*_cat_/*K*_m_ for casein and Sid4 but had a smaller effect on Rec11 (Fig. 6B). This demonstrates that CK1 autophosphorylation state can influence not only the absolute values for *k*_cat_/*K*_m_ but also the relative values of substrates compared to each other. These results suggest that in addition to direct, affinity-based competition (Fig. 3, C and D), the C-terminus may influence substrate specificity through another unknown mechanism.

Furthermore, different kinases had different catalytic efficiencies for the same substrate. Comparing Hhp2 to Hhp1, full-length autophosphorylated Hhp2 had a preference for casein over Rec11 and Sid4 (Fig. 6C). Removing Hhp2 autophosphorylation generated a pattern that resembled that of Hhp1 (Fig. 6, B and C), indicating that the two kinase isoforms have different substrate specificities when they are autophosphorylated, and these differences are diminished in the dephosphorylated forms. Similarly, for the human enzymes, the catalytic efficiencies differed between substrates, kinases, and phosphostates (Fig. 6, D and E). Consistent with previous data, removal of the CK1δv1 C-terminus greatly increased Degron phosphorylation (*34*, *35*), and the effect was more muted in CK1ε. Fully phosphorylated CK1δv1 had greater preference for casein and LRP6, while CK1ε had greater preference for LRP6 and Degron. Dephosphorylation of both enzymes tended to increase catalytic efficiencies, and the dephosphorylated forms were more similar to each other, although CK1δ retained a preference for LRP6 (Fig. 6, D and E).

To determine whether CK1 autophosphorylation state might more broadly alter the landscape of preferred substrates, we used a peptide library to interrogate the linear substrate motifs (*36*) of autophosphorylated full-length CK1δ, pT220 autophosphorylated CK1δΔC, and CK1δΔC-T220N, which is incapable of autophosphorylating any sites. pT220 CK1δΔC was isolated from unphosphorylated CK1δΔC using cation exchange chromatography and confirmed by blotting with the pT220 antibody (see Materials and Methods for details). The motif for autophosphorylated full-length CK1δ was extremely focused on −3 pT (Fig. 6F), which is consistent with the canonical CK1 motif (*3*) and with previous CK1δ peptide arrays (*36*, *37*). Truncation of the C-terminus, however, relaxed the motif for CK1δΔC (Fig. 6G). Without C-terminal autophosphorylation, preference for pT could also be seen in the −2 and −4 positions, as well as D at −3 rather than just pT. pY upstream at +2 and +3 also emerged. This suggests that when the C-terminus is dephosphorylated, substrates with a wider variety of surrounding residues may be phosphorylated, while when the C-terminus is autophosphorylated, only specific substrates exhibiting the canonical motif and with the highest affinities are targeted. Full-length autophosphorylated CK1δ also displayed a preference for a −4 G that was lost in CK1δΔC, perhaps indicating that substrates require structural flexibility to be phosphorylated in the presence of the C-terminus. In comparison to CK1δΔC, mutation of the kinase domain site in CK1δΔC-T220N had minimal effect on the peptide substrates profiled in this experiment (Fig. 6H), though a larger effect on intact protein substrates, especially LRP6 (Fig. 6D). This may indicate that in addition to the linear motif, the three-dimensional structure of substrates is playing a role in specificity.

In total, these data suggest that across yeast and human enzymes, the effects of CK1 autophosphorylation depend on each kinase-substrate pair, and autophosphorylation of both T220 in the catalytic domain and the multiple sites in the C-termini of CK1s contributes to substrate specificity.

### Phosphorylation in the kinase domain influences C-terminal autophosphorylation rate and binding affinity

**Fig. 7.**
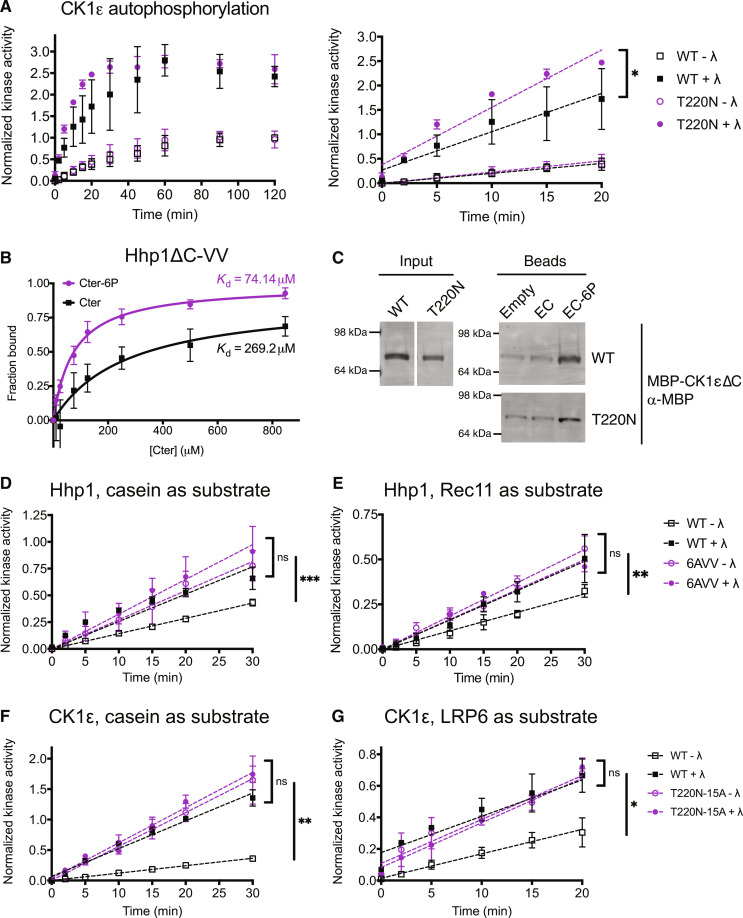
Phosphorylation in the kinase domain influences C-terminal autophosphorylation rate and binding affinity. (**A**) MBP-CK1ε was treated ± λ phosphatase (λ) and then incubated with [γ-^32^P]-ATP at 30°C. Reactions were quenched at time points from 0 to 120 min, and autophosphorylation was measured on a phosphorimager. The full time course is shown on the left; the initial rate was determined by the slope of the linear section of the curve shown on the right. (**B**) Various concentrations of Hhp1 Cter or Cter-6P beads were incubated with MBP-Hhp1ΔC-VV at 4°C for 1 hour. Protein remaining in the supernatant was visualized by Western blot and quantified by densitometry. Data from three independent replicates (mean ± SD) were fitted to a one-site specific binding curve to calculate the *K*_d_. (**C**) CK1ε EC or EC-6P was conjugated to beads and incubated with MBP-CK1εΔC at 4°C for 1 hour. Bound protein was visualized by Western blot. Representative blot from two independent replicates. Note that these data were obtained from the same experiment that was partially presented in Fig. 5E (WT versus R4A) and that the WT lanes are shown here again with the T220N lanes. (**D** and **E**) MBP-Hhp1 was treated ± λ and then incubated with substrate and [γ-^32^P]-ATP at 30°C. Reactions were quenched at time points from 0 to 120 min, and casein phosphorylation (D) or Rec11 phosphorylation (E) was measured on a phosphorimager. (**F** and **G**) MBP-CK1ε was treated ± λ and then incubated with substrate and [γ-^32^P]-ATP at 30°C. Reactions were quenched at time points from 0 to 120 min, and casein phosphorylation (F) or LRP6 phosphorylation (G) was measured on a phosphorimager. For (A) and (D) to (G), data from three independent replicates are shown as the mean ± SD. ***P < 0.0002, **P < 0.001, *P < 0.01, ns = not significant by one-way ANOVA of slopes.

In light of these findings, we asked whether T220 autophosphorylation affected the probability of tail autophosphorylation and binding to the kinase domain. Preventing kinase domain autophosphorylation by mutating the T220 site increased the rate of C-terminal autophosphorylation in Hhp1-VV (*16*) and CK1ε-T220N (Fig. 7A) compared to the wild-type kinases. The localization of C-terminal autophosphorylation sites remained constant, however, because phosphopeptide maps of Hhp1-VV and CK1ε-T220N showed no change in C-terminal phosphopeptides (figs. S1C and S6B).

Hhp1ΔC-VV and CK1εΔC-T220N were still able to bind peptides corresponding to their phosphorylated C-termini (Fig. 7, B and C). The *K*_d_ for Hhp1ΔC-VV interacting with Cter-6P was 74 μM, approximately threefold lower than for Hhp1ΔC (Fig. 2B), and we were able to detect binding of Hhp1ΔC-VV to the unphosphorylated Cter (Fig. 7B), which we had not observed for Hhp1-delC (Fig. 2B). This suggests that catalytic domain phosphorylation decreases the likelihood that the Cter will be phosphorylated and therefore also the likelihood that the Cter will interact with the kinase domain. Further, catalytic domain phosphorylation increases the selectivity for binding phosphorylated Cter by preventing promiscuous interaction of the unphosphorylated tail. When both mechanisms were simultaneously disrupted in Hhp1-VV-6A and CK1ε-T220N-15A, these mutant kinases phosphorylated their substrates at a greater rate than did the wild type, but the effects did not appear to be additive (Fig. 7, D to G), further supporting the idea that autophosphorylation in the C-terminus and autophosphorylation in the kinase domain are not independent of each other.

## DISCUSSION

Although CK1 enzymes are multifunctional kinases involved in myriad essential signaling pathways related to human disease, the mechanisms by which their catalytic activities are regulated are not fully understood. Previous research has focused on extrinsic regulation of CK1 substrates by phosphatases and priming kinases (*5*–*7*). More recent work has also shown that CK1 activity can be modulated by binding interactions (*14*, *38*), subcellular targeting (*13*, *14*), and a conserved autophosphorylation site in the kinase domain (*16*). C-terminal autophosphorylation was known to inhibit CK1 activity (*17*–*20*), and it was proposed that phosphatases are required to activate CK1 in cells (*4*). However, the biochemical model for how autophosphorylation inhibits CK1 had not been tested until recently.

We found that autophosphorylated C-termini can directly bind CK1 kinase domains to inhibit substrate phosphorylation. Our results support the idea that the C-terminus acts as a pseudosubstrate that can occupy the substrate binding groove in a manner similar to substrate peptides (*17*–*20*, *28*, *29*, *35*). Recently, binding of the CK1δ C-terminus to the kinase domain was also demonstrated by nuclear magnetic resonance (NMR) (*27*) and hydrogen-deuterium exchange mass spectrometry (*39*). In these studies, C-terminal autophosphorylation promoted interaction with the CK1δ kinase domain and inhibited phosphorylation of the substrates p63 (*27*) and PER2 (*39*). This is similar to our results using Hhp1 and CK1ε. The high substrate affinities that we measured are consistent with the product inhibition observed for p63 and the PER2 FASPS (familial advanced sleep phase syndrome) region (*28*–*30*). CK1 phosphorylates these substrates in a distributive manner, raising the questions of whether the C-termini are also phosphorylated in a distributive manner, if all sites are phosphorylated in a single molecule, and if there is any hierarchical phosphorylation of the C-termini, in which certain sites are preferentially phosphorylated.

The Cter interaction with the kinase domain has a relatively high *K*_d_ when measured by ITC (Figs. 2, B and C, and 5B). In vitro, the binding affinity was necessarily measured in trans, but in vivo, the C-terminus is attached to the kinase domain. Previous work showed that autophosphorylation of Hhp1 (*20*), rat CK1δ (*19*), human CK1α (*40*), and human CK1ε (*17*) occurs in cis. In this configuration, the Cter would be at a very high local concentration, which would favor its phosphorylation and overcome the low affinity observed in vitro. Another factor contributing to the low affinity between the C-terminus and the kinase domain could be that the autophosphorylation sites are not located in sequences that conform to the canonical CK1 motif (*3*, *36*, *41*). Other known substrates that harbor nonconsensus CK1 sites are phosphorylated at slower rates than consensus sites, for example, the FASP site on PER2 that is phosphorylated by CK1δv2 and CK1ε (*8*, *35*).

**Fig. 8.**
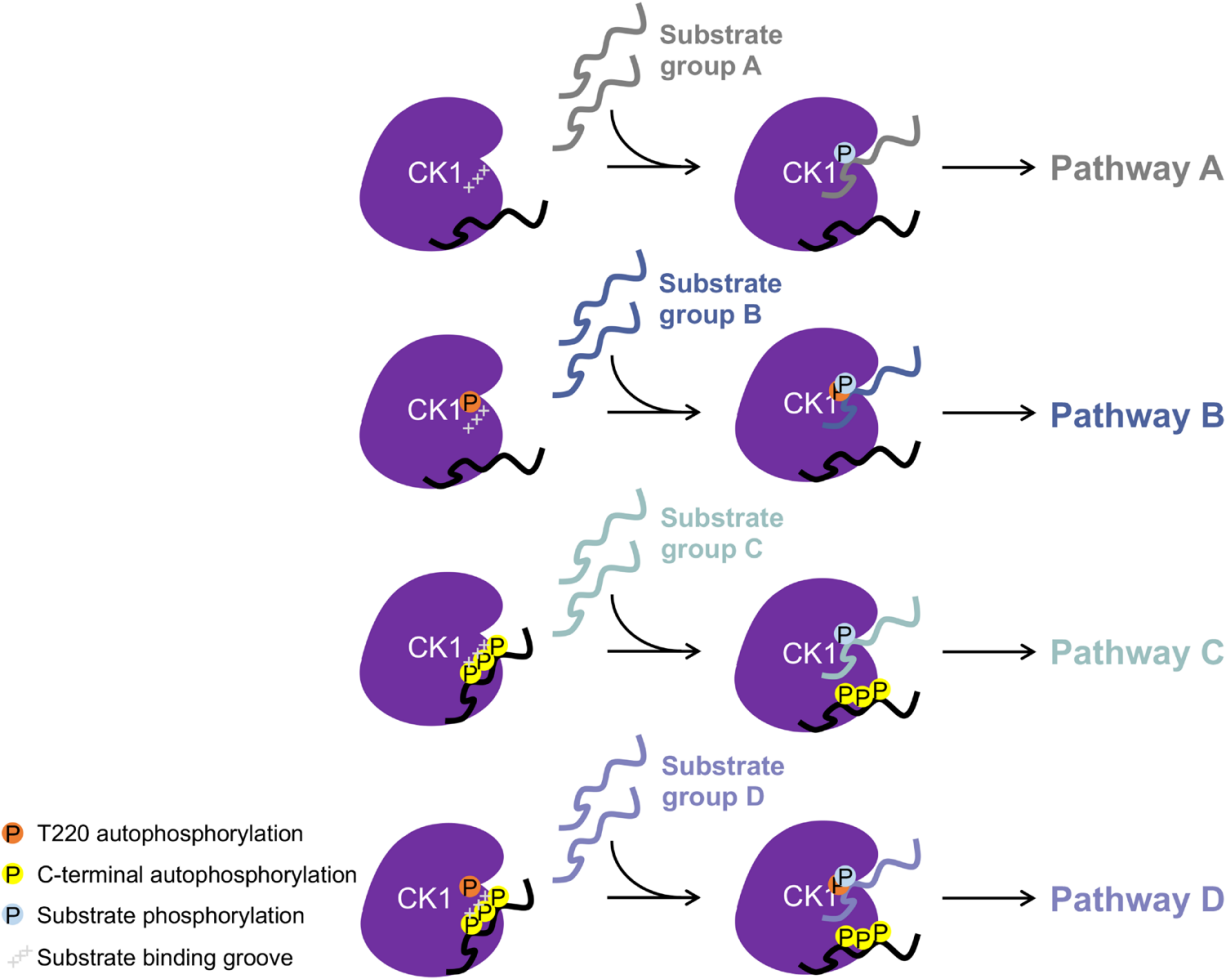
Displacement specificity model for the regulation of CK1 substrate specificity by autophosphorylation. At least four different phospho-forms of CK1 may exist. Depending on the conformation of the substrate binding groove (mediated by kinase domain autophosphorylation, orange) and the affinity of the C-terminus for the kinase domain (mediated by C-terminal autophosphorylation, yellow), different groups of substrates may be acted upon by each phospho-form (substrate phosphorylation, blue). If these substrates are involved in different signaling pathways, autophosphorylation could serve to stratify these substrates and direct CK1 activity to the proper role at the proper time.

We found that substrates have much higher affinities for the kinase domain than the phosphorylated C-terminus. Substrates can competitively inhibit Cter binding even when all autophosphorylation sites are occupied in a synthesized phosphopeptide present at very high concentration (Fig. 3, C and D). This would mean that CK1s are never fully inhibited by phosphorylated tail binding in cells, which makes biological sense if they are being used in multiple processes at multiple times. We propose a displacement specificity model (Fig. 8) in which truncation or dephosphorylation of the tail would not be strictly required for all substrate phosphorylation events. Substrates that have a higher affinity or that are present at a higher concentration may instead outcompete the tail for access to the kinase active site. In effect, the steady state between tail-bound and unbound states would be altered by the presence of substrates that have a higher affinity than the tail, increasing the proportion of active enzyme for that particular substrate. We observed competitive binding using unprimed substrates (Fig. 3, C and D), but primed substrates may potentially be even better at outcompeting the tail, and the strict motif preference observed for full-length autophosphorylated CK1δ (Fig. 6F) is consistent with this idea. The action of phosphatases on the tail may be used as an additional level of regulation to allow scarce or lower-affinity substrates to be phosphorylated under specific conditions. There is evidence that PP1 (*4*, *16*), PP2A (*4*), calcineurin (*42*), and PP5 (*43*) can dephosphorylate CK1ε to increase its activity. Future in vivo studies could address which substrates and signaling pathways require phosphatase(s) to activate CK1 and which may be independent. Furthermore, CK1 C-termini differ in length and sequence, yet they all appear to interact with the conserved kinase domain; this would imply that different tail isoforms may have different affinities for the kinase domain, allowing different substrates to displace each one. This difference in binding affinity could help explain the differences in substrate phosphorylation observed for the two splice variants of CK1δ (*8*, *39*).

In addition to affinity-based competition between the phosphorylated C-terminus and substrates, a displacement specificity model can account for other mechanisms of regulating substrate specificity, both known and unknown. The conformational changes in the substrate binding groove induced by autophosphorylation in the catalytic domain (*16*) are one such mechanism to consider. There are at least four different phospho-forms of CK1 based on occupancy of catalytic domain and tail autophosphorylation sites, each of which may have a pool of preferred substrates, so this may be one way to stratify substrates (Fig. 8). The observation that the *k*_cat_/*K*_m_ of substrates changed relative to each other depending on the CK1 phospho-form (Fig. 6, B to E) suggests that the C-terminus may influence substrate specificity by another mechanism that is not yet understood. For example, the C-terminus could affect the activity of the kinase domain through an allosteric conformational change. Conformational changes due to allosteric interactions and posttranslational modifications would be encompassed within the different phospho-forms, and it will be exciting to discover additional features of how the structure, catalytic activity, and biological function of CK1 enzymes can be modulated. We emphasize that the model presented here is incomplete, and we hope that approaching CK1 regulatory mechanisms through a lens in which different CK1 phospho-forms tune substrate specificity will facilitate future experiments that reveal additional insights into this important kinase family.

Within cells, phosphatases and other kinases also target CK1s to affect their functions (*44*–*47*). We have demonstrated that phosphorylation of yeast Hhp1 changes under different cellular stress conditions (Fig. 1, E to G and fig. S3D) and affects survival (fig. S3E); it will be informative to understand how CK1s are phosphorylated and dephosphorylated in additional organisms, cell types, and environmental conditions. This displacement specificity model provides a framework for further investigation of these aspects of CK1 function by integrating biochemical mechanism with cellular signaling outputs, and it can be continually tested and refined as we uncover more about CK1 signaling.

## MATERIALS AND METHODS

### Molecular biology and protein purification

All plasmids were generated by standard molecular biology techniques. For protein production, cDNA for Hhp1, Hhp2, CK1δv1, and CK1ε was cloned into the pMAL-C2 vector. CK1ΔC constructs consisted of the following amino acids: Hhp1 1–296, Hhp2 1–295, CK1δ 1–294, and CK1ε 1–294. Mutagenesis was performed using QuikChange XL and QuikChange Multi site-directed mutagenesis kits (Agilent Technologies). Plasmids were validated by DNA sequencing.

Protein production was induced in *Escherichia coli* Rosetta2(DE3)pLysS cells by addition of 0.1 mM isopropyl-β-D-thiogalactopyranoside (IPTG) overnight at 17°C. Cells were lysed using lysozyme (300 μg/ml) for 20 min followed by sonication. MBP fusion proteins were purified on amylose beads (New England Biolabs) in column buffer [20 mM tris (pH 7.4), 150 mM NaCl, 1 mM EDTA, 0.1% NP-40, 1 mM dithiothreitol (DTT), 1 mM phenylmethylsulfonyl fluoride (PMSF), 1.3 mM benzamidine, protease inhibitor tablets (Roche)] and eluted with maltose [20 mM tris (pH 7.4), 150 mM NaCl, 1 mM EDTA, 1 mM DTT, 1 mM PMSF, 1.3 mM benzamidine, 10 mM maltose, 10% glycerol]. For purification of Sid4, Sid4 cDNA was cloned into the pET His6 MBP PreScission LIC cloning vector (gift from S. Gradia, Addgene plasmid #29721). His-MBP-Sid4 was purified on amylose beads in column buffer containing 300 mM NaCl, 20 mM MgCl_2_, and 20 mM Na_2_SO_4_. On-bead cleavage with PreScission (GE Healthcare) protease was performed in cleavage buffer [50 mM tris (pH 7.0), 300 mM NaCl, 1 mM EDTA, 0.1% NP-40, 1 mM DTT, 10% glycerol, 20 mM MgCl_2_, 20 mM Na_2_SO_4_] for 16 hours at 4°C using 1 U protease per 200 μg of fusion protein. Protease was removed by incubation with GST-Bind beads (EMD Millipore). Sid4 was concentrated using Amicon Ultra centrifugal filters (Millipore). GST fusion proteins were purified on GST-Bind beads in GST Bind buffer [10 mM NaPO_4_ (pH 7.3), 150 mM NaCl, 2.7 mM KCl, 0.1% NP-40, 1 mM EDTA, 1 mM DTT, 1 mM PMSF, 1.3 mM benzamidine, protease inhibitor tablets (Roche)] and eluted with glutathione [50 mM tris (pH 8.0), 100 mM NaCl, 1 mM DTT, 1 mM PMSF, 1.3 mM benzamidine, 10 mM glutathione, 10% glycerol]. The human PER2 Degron sequence (amino acids 475 to 505) (*29*) was synthesized as a gBlock (IDT), cloned into pMAL-C2, and expressed and purified as above. GST-Rec11-N3 and MBP-LRP6ΔN were expressed and purified as previously described (*16*). The casein used as a CK1 substrate was dephosphorylated alpha casein (Sigma, product #C8032). 

### In vitro kinase assays

Kinases were treated with 0.5 μl of λ phosphatase (New England Biolabs) per 1 μg of protein for 45 min at 30°C in Protein MetalloPhosphatase (PMP) buffer (New England Biolabs) plus 1 mM MnCl_2_. For negative controls, an equivalent volume of buffer was added instead of phosphatase. Phosphatase reactions were quenched by the addition of 8 mM Na_3_VO_4_ or PhosSTOP (Roche) immediately before the kinase assay. All kinase assays were performed at 30°C and quenched by boiling in SDS-PAGE sample buffer. Proteins were separated by SDS-PAGE and stained in Coomassie to visualize total protein, and then gels were dried before detection of phosphoproteins.

Phosphorylation of the Hhp1 and CK1ε Cter was performed with 1 μg of MBP-Hhp1ΔC or 1 μg of MBP-CK1εΔC and 4 μg of GST-Hhp1 Cter or GST-CK1ε Cter in PMP buffer plus 250 μM cold adenosine triphosphate (ATP), 1 μCi of [γ-^32^P]-ATP, and 10 mM MgCl_2_ for 2 hours. Phosphorylation of casein and Rec11 by Hhp1 and LRP6 by CK1ε mutants was performed with 300 ng of kinase and 25 μM substrate in PMP buffer plus 100 μM cold ATP, 1 μCi of [γ-^32^P]-ATP, and 10 mM MgCl_2_ for 45 min. Phosphorylated proteins were visualized by autoradiography.

Phosphorylation of casein and Rec11 in the presence of Hhp1 Cter and LRP6 in the presence of CK1ε Cter was performed with 200 ng of MBP-Hhp1ΔC or MBP-CK1εΔC, 25 μM substrate, and 0 to 455 μM Cter peptide in PMP buffer plus 250 μM cold ATP, 1 μCi of [γ-^32^P]-ATP, and 10 mM MgCl_2_ for 1 hour. Kinetic assays were performed using 200 ng of MBP-Hhp1 or MBP-CK1ε and 25 μM substrate in PMP buffer plus 250 μM cold ATP, 1 μCi of [γ-^32^P]-ATP, and 10 mM MgCl_2_. Reactions were quenched at time points from 0 to 120 min. Phosphorylated proteins were visualized using an FLA7000IP Typhoon Storage Phosphorimager (GE Healthcare Life Sciences) and quantified in ImageJ. For kinetics assays in Figs. 1 (C and D), 4 (C and D), and 7 (A and D to G) and fig. S2 (A and B), the signal for each kinase at each time point was normalized to the signal for WT − λ at 120 min. For assays in Figs. 3 (A and B) and 5C, the signal at each concentration of Cter was normalized to the signal at 0 μM. Relative kinase activity ratios were plotted in Prism.

To determine the *k*_cat_/*K*_m_, 200 ng of MBP-tagged kinase (0.12 μM for full-length and 0.13 μM for ΔC) and 0.62 μM substrate were incubated in PMP buffer plus 250 μM cold ATP, 2 μCi of [γ-^32^P]-ATP, and 10 mM MgCl_2_. For the experiments in Fig. S7C, 400 μM pCter was added in addition to 0.13 μM Hhp1ΔC. Reactions were quenched at time points from 0 to 60 min. Phosphorylated proteins were visualized using an FLA7000IP Typhoon Storage Phosphorimager, quantified in ImageJ, and scaled to the moles of substrate. At this substrate concentration, [*S*] << *K*_m_ and [*E*]_free_ approximately equals [*E*]_T_. Therefore, the measured reaction rate (*V*_o_) equals (*k*_cat_/*K*_m_)[*E*]_T_[*S*].

### In vitro binding assays

Peptides corresponding to amino acids 297 to 365 of Hhp1 were synthesized and linked to biotin at the N terminus, either unphosphorylated (Cter) or phosphorylated at S326, S327, T345, T346, S354, and T356 (Cter-6P), by Bio-Synthesis Inc. Peptides corresponding to amino acids 383 to 416 of CK1ε were synthesized and linked to biotin at the N terminus, either unphosphorylated (EC) or phosphorylated at S389, S390, S391, S405, T407, and S408 (EC-6P), by LifeTein LLC. Peptides were conjugated to streptavidin sepharose (Amersham Biosciences) at a concentration of 6 μg of peptide per 1-μl bead slurry by rotating at 4°C overnight in binding buffer [50 mM tris (pH 7.0), 25 mM NaCl, 1 mM EDTA, 1 mM DTT]. Beads were washed to remove unbound peptide, and the concentration of bound peptide was confirmed by SDS-PAGE. In all experiments, unconjugated streptavidin sepharose was used as a negative control (“Empty”), and beads were blocked in 5% bovine serum albumin (BSA) by rotating at 4°C for 30 min before binding assays to reduce nonspecific binding.

Peptide pull-downs were performed by incubating 2 μg of MBP-Hhp1ΔC or MBP-CK1εΔC with 500 μM peptide in binding buffer for 1 hour at 4°C, rotating. Beads were washed to remove unbound kinase and then boiled in SDS-PAGE sample buffer to elute. Bound protein was detected by Western blot using mouse anti-MBP diluted 1:10,000 (New England Biolabs), fluorescent secondary antibodies (Li-Cor Biosciences), and an Odyssey CLx (Li-Cor Biosciences).

Equilibrium binding assays were performed according to (*48*): The volume of peptide-conjugated beads was adjusted to yield peptide concentrations of 0 to 847 μM in the final reaction volume of 50 μl. Unconjugated streptavidin sepharose was then added to equalize the bead volume of all reactions. Beads were incubated with 100 ng of MBP-Hhp1ΔC or 50 ng of MBP-CK1εΔC in binding buffer for 1 hour at 4°C, rotating. The supernatants were removed, and unbound kinase was detected by Western blot as above. The unbound fraction was quantified on the Odyssey CLx, background-subtracted, and normalized to the 0 μM condition. The fraction bound (1 − unbound) was plotted in Prism and fit to a one-site specific binding equation to calculate the *K*_d_. Competitive binding experiments were performed as above, but 25 μM substrate was added to the beads along with the kinase. The one-site specific binding equation was constrained such that B_max_ = 1 to calculate the apparent *K*_d_. The *K*_i_ (inhibition constant) was determined by the equation below, where [*I*] = [substrate] = 25 μM:

$$K_{d\left(\text{app}\right)}=K_{d}\left(1+\frac{\left[I\right]}{K_{i}}\right)$$

### Isothermal titration calorimetry

ITC measurements were conducted on a Nano-ITC instrument (TA Instruments) in ITC buffer [10 mM NaPO_4_ (pH 7.5), 150 mM NaCl, 0.25 mM tris(2-carboxyethyl)phosphine (TCEP)]. The sample cell was filled with 300 μl of 100 μM MBP-Hhp1ΔC or MBP-CK1εΔC, while the syringe contained 50 μl of 2 mM peptide (Cter, Cter-6P, EC, or EC-6P). All solutions were degassed before being loaded into the cell. Aliquots (2 μl) of peptide solutions were injected into CK1 protein solutions at 25°C with an interval gap of 200 s and the syringe rotating at 150 rpm to ensure proper mixing. Data were analyzed using Nanoanalyser software to extract the thermodynamic parameters; the *K*_d_ was obtained after fitting the integrated and normalized data to a single-site binding model. Experiments were performed in duplicate to ensure reproducibility (see Figs. 2C and 5B and fig. S4, A to D).

### Yeast methods

*S. pombe* strains used in this study (table S1) were grown in yeast extract (YE) medium (*49*). For *hhp1* gene replacements, haploid *hhp1::ura4+* was transformed using standard lithium acetate methods (*50*) to introduce linear *hhp1-6A* and *hhp1-6N* gene fragments generated by digestion of pIRT2-hhp1-6A and pIRT2-hhp1-6N plasmids with Bam HI and Pst I. Integrants were selected based on resistance to 5-fluoroorotic acid (1.5 mg/ml) (Fisher Scientific) and validated by whole-cell polymerase chain reaction (PCR) using primers homologous to endogenous flanking sequences in combination with those within the ORF. Truncation of *hhp1* was accomplished by insertion of the *kanMX6* cassette from pFA6 as previously described (*51*), followed by selection on G418 (100 μg/ml; Sigma-Aldrich, St. Louis, MO). Tagged Hhp1-6N was generated by insertion of the *3xFLAG:kanMX6* cassette from pFA6 as previously described (*51*), followed by selection on G418. Insertions were validated by whole-cell PCR using primers homologous to the resistance cassette and the endogenous open reading frame (ORF). Diploid strains were constructed using standard techniques (*49*); intragenic complementation between *ade6-M210* and *ade6-M216* alleles and maintenance on *ade^−^* growth medium ensured that strains remained stable. All constructs and integrants were sequenced to verify their accuracy. *S. pombe* genomic sequences and annotation were from PomBase (*52*).

For serial dilution growth assays, cells were cultured in YE at 29°C or 32°C until mid-log phase, three 10-fold serial dilutions starting at 4 × 10^6^ cells/ml were made, 2.5 μl or 4 μl of each dilution was spotted on YE plates or YE plates containing HU (Sigma-Aldrich, St. Louis, MO), and cells were grown at the indicated temperatures for 2 to 5 days.

For immunoprecipitations (IPs), cell pellets were snap-frozen and then lysed by bead disruption using a FastPrep cell homogenizer (MP Biomedicals) in NP-40 buffer under denaturing conditions as previously described (*53*), except with the addition of protease inhibitor tablets (Roche) and phosphatase inhibitor tablets (Roche). Hhp1-3xFLAG was immunoprecipitated using 2 μg of FLAG-M2 (Sigma). Proteins were separated by SDS-PAGE, transferred to Immobilon-P polyvinylidene fluoride membrane (Millipore), and immunoblotted with FLAG-M2 (1 μg/ml) followed by fluorescent anti-mouse secondary antibody (Li-Cor Biosciences) and imaging on an Odyssey CLx (Li-Cor Biosciences). Phos-Tag gels used 40 μM Phos-Tag reagent and 6% acrylamide. Cells were incubated in 12 mM HU for 3.5 hours before IP. Cells were exposed to 44°C for 0, 0.5, 1, or 2 hours before IP.

Heat shock survival assay was done as described previously (*16*, *54*). Briefly, cells were grown in YE at 29°C to mid-log phase. Cells were then split into two flasks, with one to continue to incubate at 29°C (non–heat shock) and the other to incubate at 44°C (heat shock) for 3 hours. Two hundred cells from either condition were plated onto YE in triplicate and incubated at 29°C for 6 days. Colonies were counted and normalized to the non–heat shock condition to score percent viability.

### Mass spectrometry

Trichloroacetic acid (TCA)-precipitated proteins were subjected to mass spectrometric analysis on an LTQ Velos (Thermo Fisher Scientific) by three-phase multidimensional protein identification technology (MudPIT) as previously described (*55*) with the following modifications. Proteins were resuspended in 8 M urea buffer (8 M urea in 100 mM tris, pH 8.5), reduced with tris (2-carboxyethyl) phosphine, alkylated with 2-chloro acetamide, and digested with trypsin or elastase. The resulting peptides were desalted by C-18 spin column (Pierce). For the kinase assay samples, six salt elution steps were used (i.e., 25, 50, 100, 600, 1000, and 5000 mM ammonium acetate). Raw mass spectrometry data were filtered with Scansifter and searched by SEQUEST. Scaffold (version 3.6.0 or version 4.2.1) and Scaffold PTM (version 3.0.1) (both from Proteome Software, Portland, OR) were used for data assembly and filtering. The following filtering criteria were used: minimum of 90.0% peptide identification probability, minimum of 99% protein identification probability, and minimum of two unique peptides.

### Phosphopeptide mapping

Autophosphorylation reactions were performed with 4 μg of λ phosphatase–treated kinase in PMP buffer plus 100 μM cold ATP, 4 μCi of [γ-^32^P]-ATP, and 10 mM MgCl_2_ at 30°C for 30 min. Reactions were quenched by boiling in SDS-PAGE sample buffer, and proteins were separated by SDS-PAGE. Phosphorylated proteins were transferred to polyvinylidene difluoride (PVDF) membranes. Proteins were digested off the membrane with 10 μg of trypsin at 37°C overnight. Peptides were lyophilized and resuspended in pH 1.9 buffer. Tryptic peptides were separated in the first dimension by thin-layer electrophoresis and in the second dimension by chromatography (*56*). After separations, thin-layer chromatography (TLC) plates were exposed to film for 2 to 4 days at −80°C with intensifying screens.

### Circular dichroism

Recombinant MBP-Hhp1ΔC and MBP-CK1εΔC proteins were dialyzed into circular dichroism (CD) buffer [10 mM sodium phosphate (pH 7.4), 150 mM Na_2_SO_4_, 0.5 mM EDTA, 0.5 mM DTT] overnight at 4°C. Proteins were diluted to 100 ng/μl in CD buffer and measured at room temperature in a 1-mm path length cuvette on a Jasco J-810 spectropolarimeter. Far-UV measurements were collected over a range of 260 to 190 nm at 0.5-nm resolution, and three scans were averaged for each spectrum. A blank consisting of CD buffer was subtracted from each protein spectrum, and the data (mdeg) were converted to mean residue ellipticity.

### Linear substrate motif determination by peptide library

Reagents used for the peptide library experiments included the kinase substrate library (AnaSpec) and streptavidin-conjugated membranes (Promega). CK1δ full-length was obtained from Thermo Fisher Scientific (catalog no. PV3987). cDNA for CK1δ∆C was cloned into pET28-MBP-TEV (Addgene #69929); this construct was also mutagenized to introduce T220N. His_6_-MBP-CK1δ∆C WT and T220N were expressed as described above, purified using a HisTrap HP column (Cytiva) in binding buffer containing 50 mM tris (pH 7.5), 500 mM NaCl, 30 mM imidazole, and 5% glycerol, and then eluted with elution buffer containing 50 mM tris (pH 7.5), 500 mM NaCl, 300 mM imidazole, and 5% glycerol. To ensure maximal autophosphorylation of T220, the eluted His_6_-MBP-CK1δ∆C WT was incubated in PMP buffer containing 10 mM MgCl_2_ and 100 μM ATP at 4°C overnight. His_6_-MBP-CK1δ∆C WT and T220N were then dialyzed against MBP column buffer and cleaved with TEV protease at 4°C overnight. The tag was removed using MBPTrap HP (Cytiva), and CK1δ∆C WT and T220N were concentrated using Amicon Ultra centrifugal filters. To separate pT220-CK1δ∆C from remaining unphosphorylated protein, cation exchange was performed using a Capto HiRes S column (Cytiva) on the CK1δ∆C WT protein. Protein was diluted in buffer containing 50 mM tris (pH 8.8) and 0.5 mM TCEP (1:20, v/v) and eluted using a salt gradient from 0 to 1 M NaCl. The isolated pT220-CK1δ∆C was pooled and buffer-exchanged with buffer containing 50 mM tris (pH 8.0), 100 mM NaCl, 2 mM MgCl_2_, and 2% glycerol, and then 1 mM TCEP and 25% glycerol were added before freezing. Phosphorylation at T220 was confirmed by Western blotting using anti-pT220 antibody (*16*).

To determine the substrate motifs, we performed in vitro phosphorylation assays with recombinant kinases on an oriented peptide array library of design Y-A-X-X-X-X-X-[S/T]-X-X-X-X-X-G-K-K-biotin in the presence of [γ-^32^P]-ATP (*36*, *37*). Reactions were performed in buffer containing 50 mM Hepes (pH 7.5), 100 mM NaCl, 2 mM DTT, 0.01% Brij 35, 10 mM MgCl_2_, 20 μM ATP, and 0.4 μCi of (33 nM) [γ-^32^P]-ATP at 30°C for 90 min. The peptides were spotted onto streptavidin-coated filter sheets and visualized by phosphorimaging on a Typhoon 5 (GE Healthcare Life Sciences).

## Supplementary Materials

### This PDF file includes:

Figs. S1 to S7 Table S1 References
