# Supplementary material for: Substrate displacement of CK1 C-termini regulates kinase specificity
Source: Sci Adv. 2024 May 10;10(19):eadj5185. doi: 10.1126/sciadv.adj5185 (PMC11086627; doi:10.1126/sciadv.adj5185)
Supplement: Supplementary file 1 — Figs. S1 to S7 Table S1 References [file sciadv.adj5185_sm.pdf]

Science Advances
AAAS

Supplementary Materials for

Substrate displacement of CK1 C-termini regulates kinase specificity

Sierra N. Cullati *et al.*

Corresponding author: Kathleen L. Gould, kathy.gould@vanderbilt.edu

Sci. Adv. **10**, eadj5185 (2024)
DOI: 10.1126/sciadv.adj5185

This PDF file includes:

Figs. S1 to S7
Table S1
References

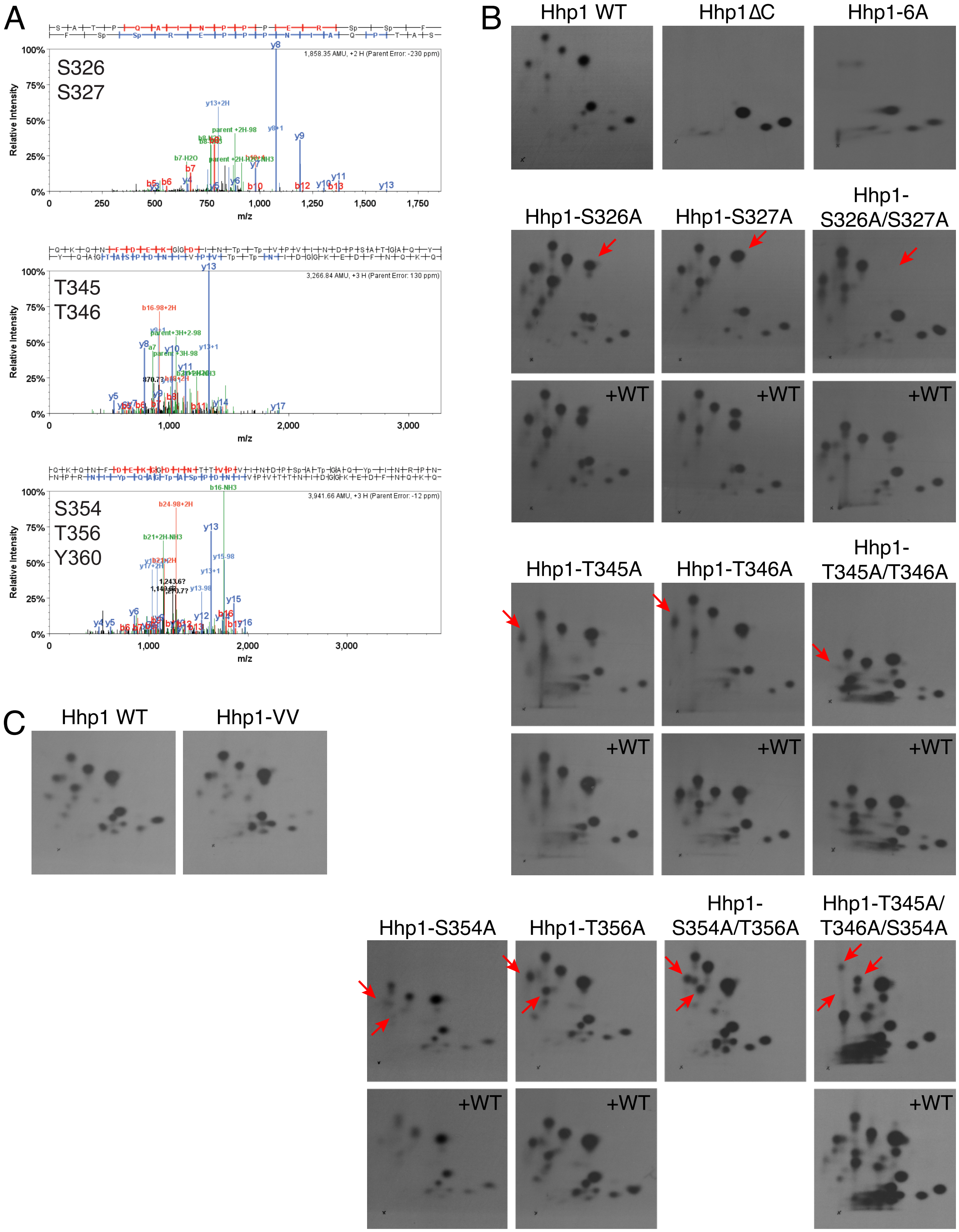

Supplemental Figure 1

Figure S1: Identification of Hhp1 autophosphorylation sites. (A) Recombinant MBP-Hhp1 was analyzed for post-translational modifications by mass spectrometry. Representative spectra of candidate autophosphorylation sites are shown. (B) The indicated Hhp1 mutants was treated with lambda phosphatase, then incubated with γ-[^{32}P]-ATP at 30°C for 30 min. Proteins were digested with trypsin, and peptides were separated by thin-layer electrophoresis and chromatography. Phosphopeptides were detected by autoradiography. Red arrows point out phosphopeptides affected by alanine substitutions. (C) Phosphopeptide map of C-terminal autophosphorylation sites in MBP-Hhp1-VV.

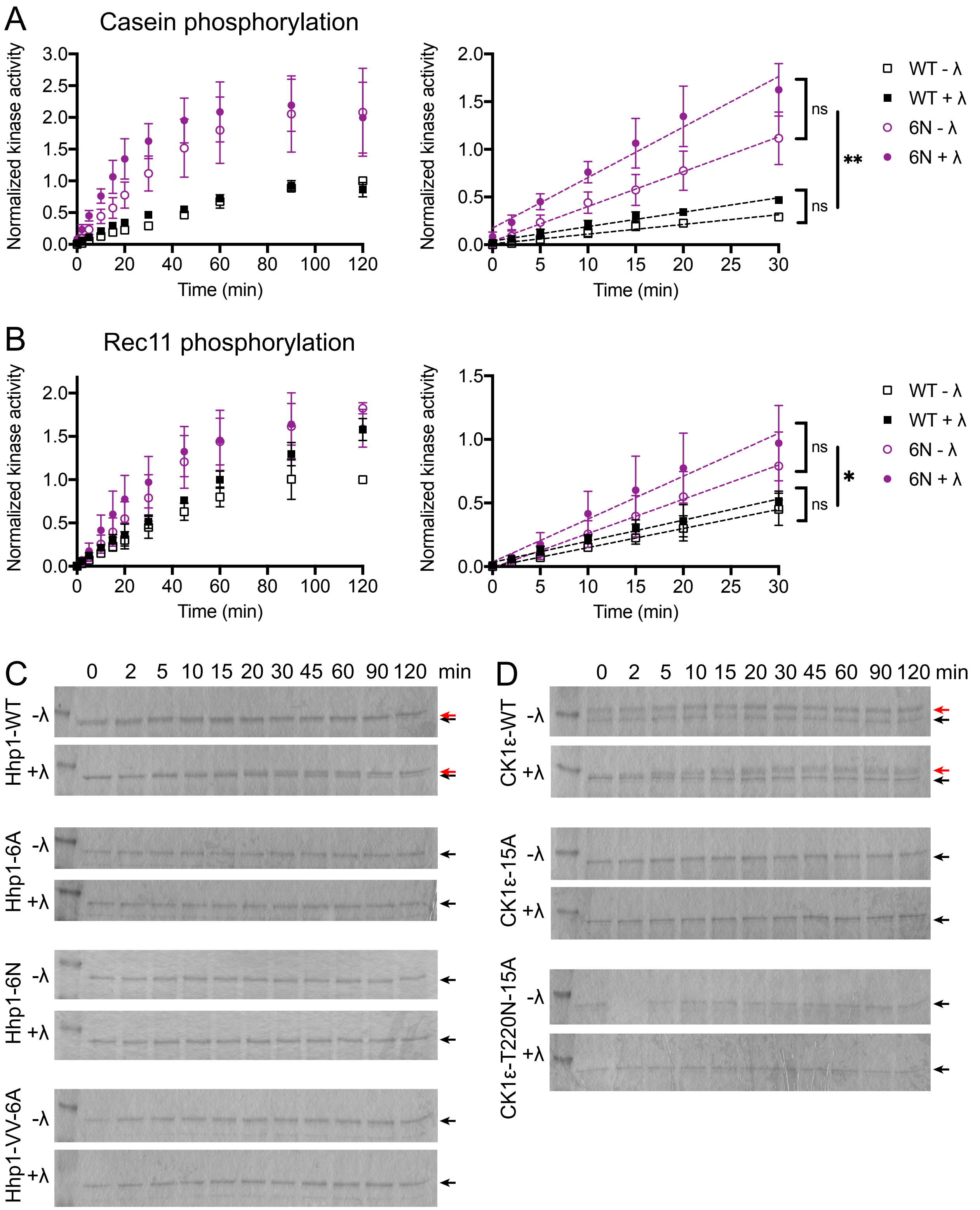

Supplemental Figure 2

Figure S2: Substrate phosphorylation kinetics of Hhp1-6N and autophosphorylation of Hhp1 and CK1ε. (A-B) MBP-Hhp1-6N was treated +/- lambda phosphatase (λ), then incubated with substrate and γ-[^{32}P]-ATP at 30°C. Reactions were quenched at timepoints from 0-120 min, and casein phosphorylation (A) or Rec11 phosphorylation (B) was measured on a phosphorimager. Data from three independent replicates is shown as the mean ± SD. *** = $p < 0.0002$, ** = $p < 0.001$, ns = not significant by one-way ANOVA of slopes. (C-D) Representative Coomassie-stained SDS-PAGE gels demonstrating the phosphorylation-dependent mobility shift of Hhp1 (C) and CK1ε (D) that occurs during kinetics experiments shown in Fig. 1 and Fig. 4. When the C-termini are able to be phosphorylated, the initially unphosphorylated kinase (black arrows) is quickly but not completely converted to a slower migrating species (red arrows).

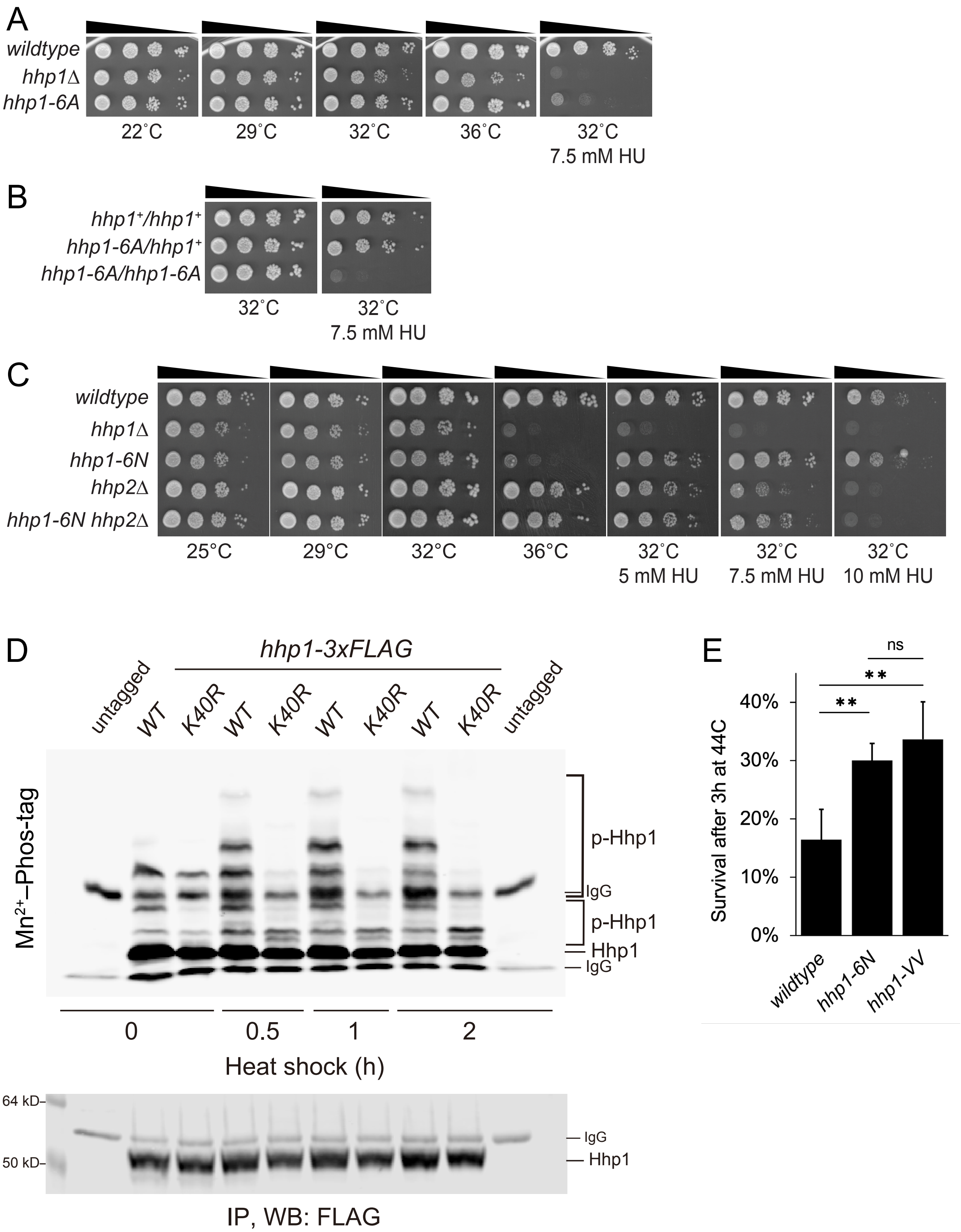

Supplemental Figure 3

Figure S3: Characterization of *hhp1* C-terminal autophosphorylation site mutants in vivo. (A-C) Mutant alleles were integrated at the endogenous *hhp1* locus, and 10-fold serial dilutions were spotted on YE with and without the indicated concentrations of hydroxyurea (HU). Cells were grown for 2-5 d at the indicated temperatures. Representative plates from 3 independent replicates are shown. (B) Diploid strains demonstrating *hhp1-6A* recessive loss-of-function. (D) *hhp1-wt* and *hhp1-K40R* were tagged with 3xFLAG and heat-shocked at 44°C for 0, 0.5, 1, or 2 h. The resultant proteins were immunoprecipitated from denatured lysates with anti-FLAG antibody and electrophoretic mobility shifts on 6% gels containing 40 μM Mn^{2+}–Phos-Tag were detected with anti-FLAG antibody. (E) Heat shock assay demonstrating *hhp1-6N* gain-of-function. Cells were grown to log phase at 29°C, then shifted to 44°C for 3h. Cells were plated in triplicate and scored for viability at 29°C. Data from three independent replicates is shown as the mean ± SD. ** = $p < 0.001$ by two-tailed t-test.

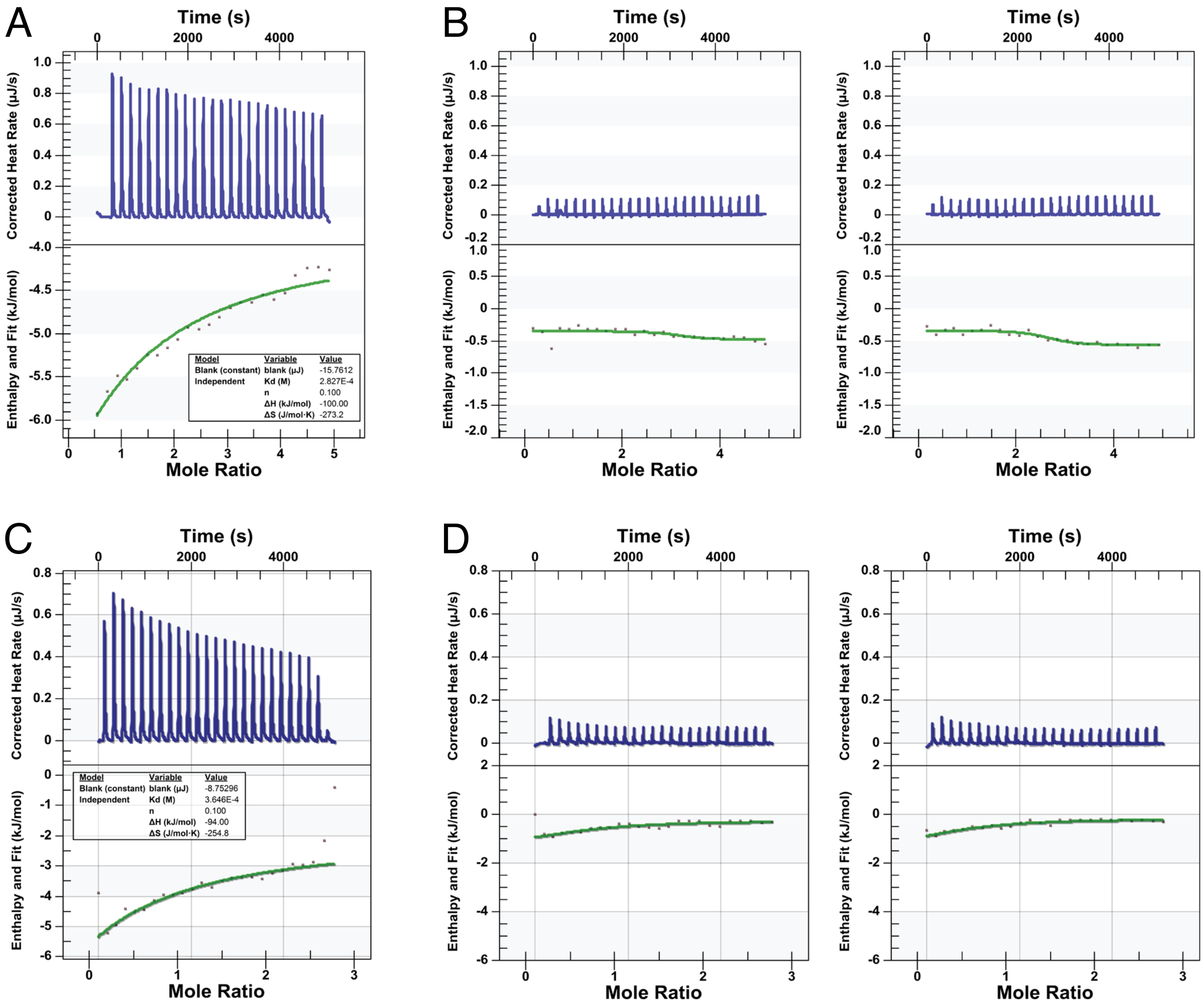

Supplemental Figure 4

Figure S4: Hhp1 and CK1ε do not bind unphosphorylated tail peptides. Binding affinities determined by ITC. Top panel shows raw data; bottom panel shows normalized integrated data. (A) Second replicate of MBP-Hhp1ΔC binding Cter-6P. (B) MBP-Hhp1ΔC incubated with Cter. (C) Second replicate of MBP-CK1εΔC binding EC-6P. (D) MBP-CK1εΔC incubated with EC.

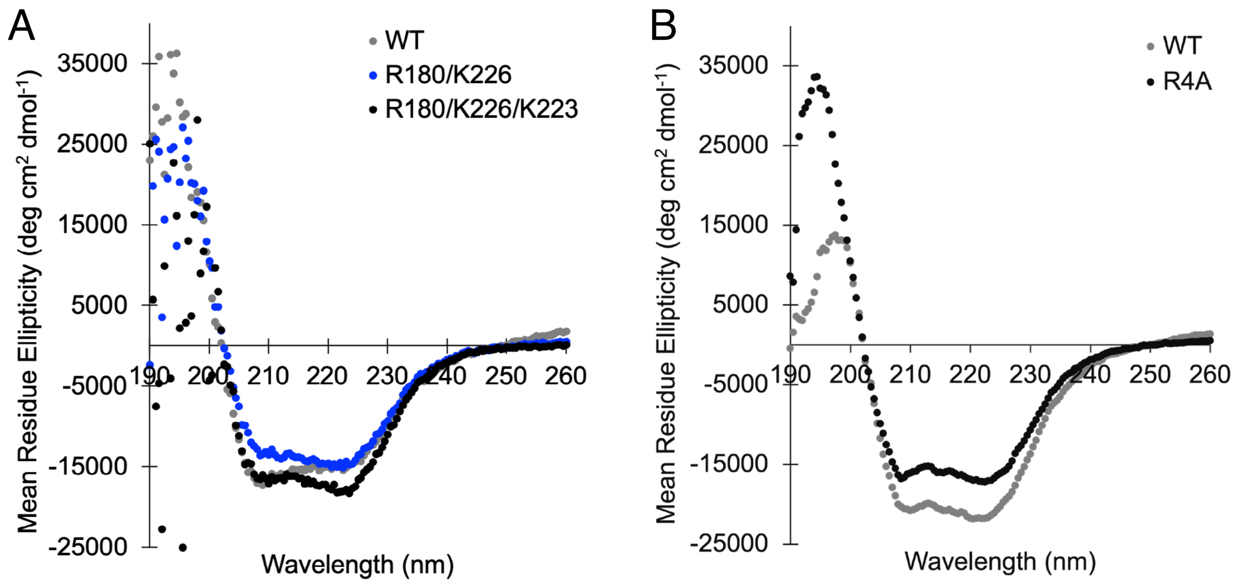

Supplemental Figure 5

Figure S5: Mutating the substrate binding groove does not disrupt overall kinase domain folding. Circular dichroism in the far-UV region of MBP-Hhp1ΔC (A) and MBP-CK1εΔC (B) wildtype and mutant proteins.

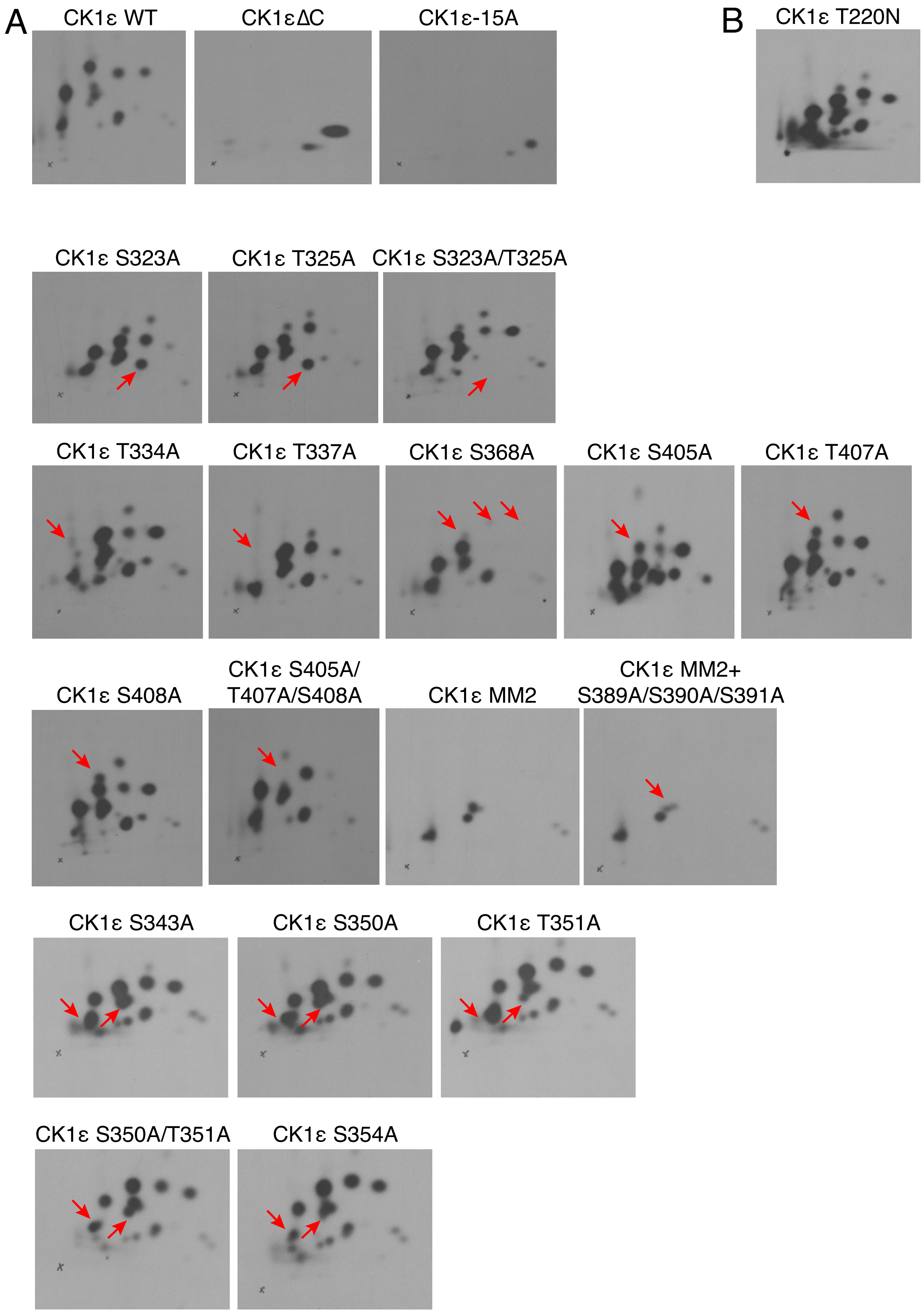

Supplemental Figure 6

Figure S6: Identification of CK1ε autophosphorylation sites. (A) The indicated CK1ε mutants were treated with lambda phosphatase, then incubated with γ-[^{32}P]-ATP at 30°C for 30 min. Proteins were digested with trypsin, and peptides were separated by thin-layer electrophoresis and chromatography. Phosphopeptides were detected by autoradiography. Red arrows point out phosphopeptides affected by alanine substitutions. CK1ε-MM2 consists of S323A, T325A, T334A, T337A, S368A, S405A, T407A, and S408A (*18*). (B) Phosphopeptide map of C-terminal autophosphorylation sites in MBP-CK1ε-T220N.

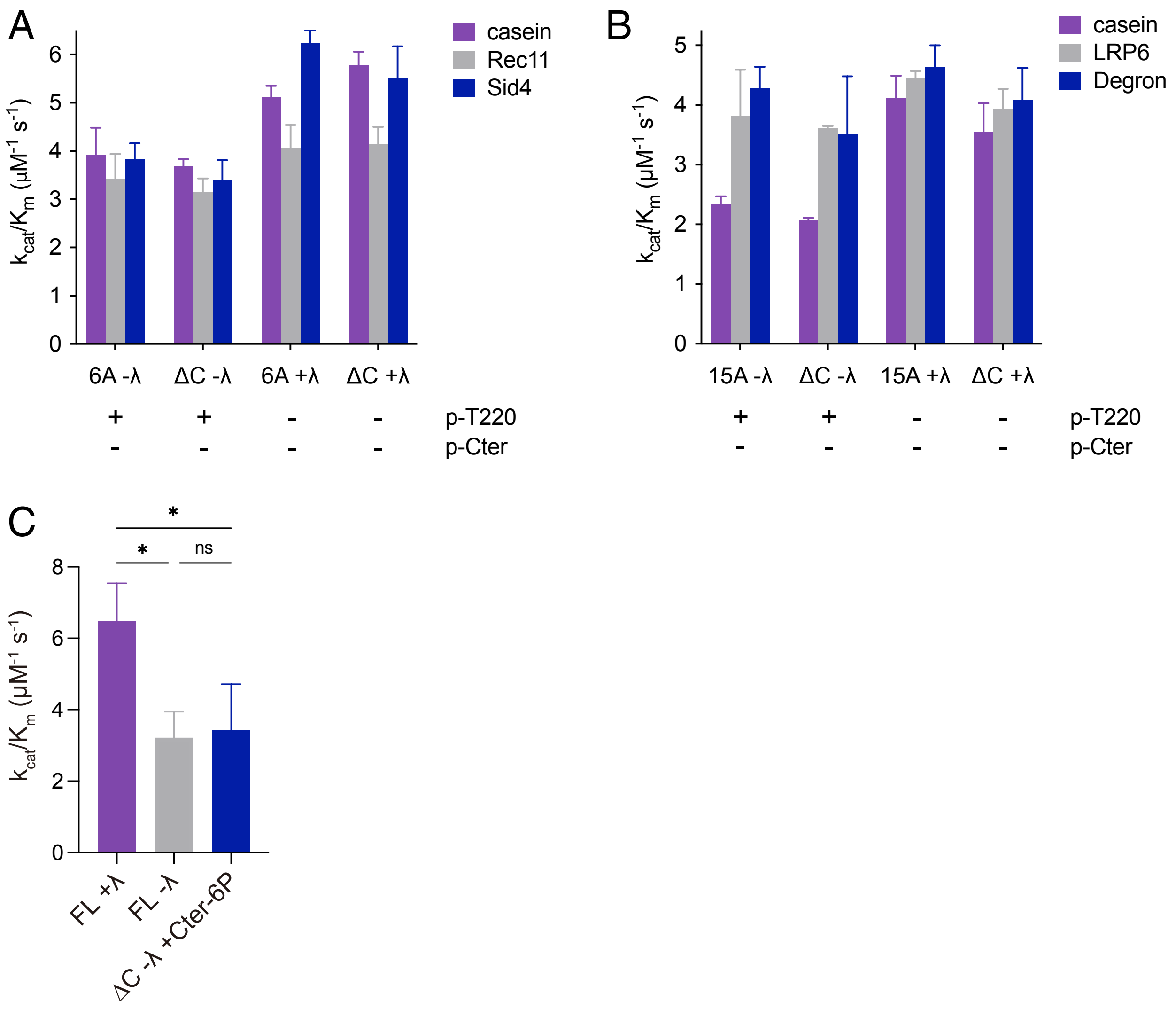

Supplemental Figure 7

Figure S7: Additional specificity footprints of Hhp1 and CK1ε. (A-C) The indicated CK1 enzymes were treated +/- lambda phosphatase (λ), then incubated with the indicated substrates and γ-[^{32}P]-ATP at 30°C. Reactions were quenched at timepoints from 0-60 min, and substrate phosphorylation was measured on a phosphorimager. The initial rate was determined by the slope of the linear section of the curve, then used to calculate the k_{cat}/K_m (see Methods for details). Data from three independent replicates is shown as the mean ± SD. (A) Specificity footprint of Hhp1-6A. (B) Specificity footprint of CK1ε-15A. (C) Catalytic efficiency of casein phosphorylation using Hhp1 FL vs Hhp1ΔC incubated with 400 μM pCter *in trans*. * = $p < 0.05$, ns = not significant by one-way ANOVA.

Supplemental Table 1: *S. pombe* strains used in this study.

Strain	Genotype	Reference
Figure 1		
246	*ade6-M210 ura4-D18 leu1-32 h-*	Lab stock
14041	*hhp1-3xFLAG:kanMX6 ura4-D18 leu1-32 ade6-M210 h-*	This study
8548-2	*hhp1-6N-3xFLAG:kanMX6 ura4-D18 leu1-32 h-*	This study
7549-2	*hhp1-K40R-3xFLAG:kanMX6 ura4-D18 leu1-32 h-*	This study
Supplemental Figure 3		
599	*ura4-D18 leu1-32 h-*	Lab stock
6415	*hhp1Δ::ura4^{+} ura4-D18 leu1-32 h-*	*(59)*
2780-2	*hhp1-6A ura4-D18 leu1-32 h-*	This study
1998-2	*hhp1-6N ura4-D18 leu1-32 h-*	This study
*	*ade6-M210/ade6-M216 ura4-D18/ura4-D18 leu1-32/leu1-32 h-/h+*	This study
*	*hhp1^{+}/hhp1-6A ade6-M216/ade6-M210 ura4-D18/ura4-D18 leu1-32/leu1-32 h-/h+*	This study
*	*hhp1-6A/hhp1-6A ade6-M210/ade6-M216 ura4-D18/ura4-D18 leu1-32/leu1-32 h-/h+*	This study
16474	*hhp1::kanMX6 ade6-M210 ura4-D18 leu1-32 h+*	Lab stock
8334-2	*hhp2::kanMX6 ura4-D18 leu1-32 h-*	This study
8329-2	*hhp1-6N hhp2::kanMX6 ura4-D18 leu1-32 h-*	This study
5106-2	*hhp1-VV ura4-D18 leu1-32 h-*	*(16)*

*Diploids were made fresh immediately prior to each experiment.

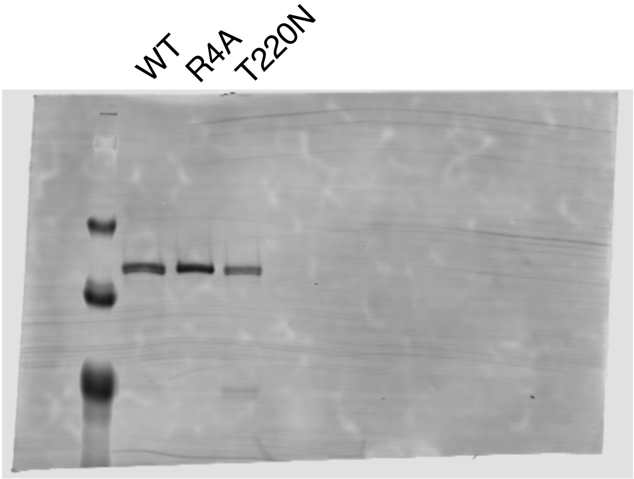

Input,
shown in Fig 5E and Fig 7C

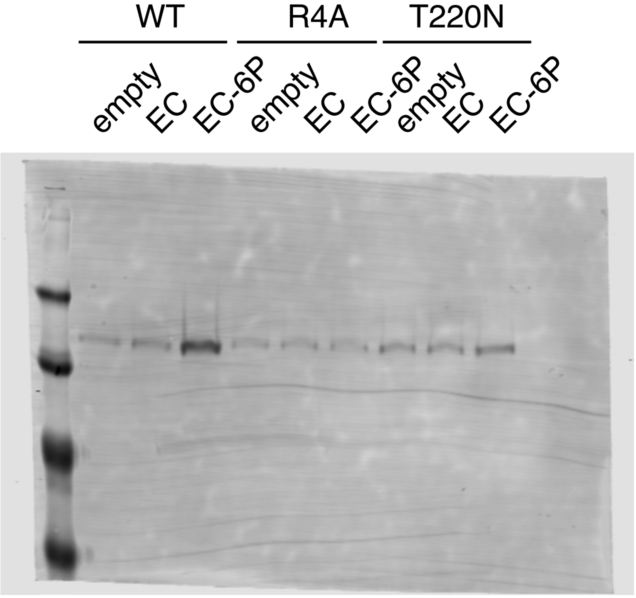

Pulldown, replicate 1,
shown in Fig 5E and Fig 7C

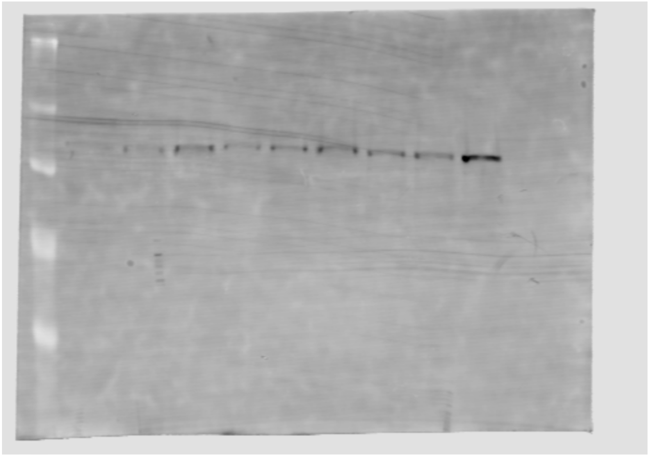

Pulldown, replicate 2,
shown in Fig 7C

Uncropped Western blots

REFERENCES AND NOTES

1. J. K. Cheong, D. M. Virshup, Casein kinase 1: Complexity in the family. *Int. J. Biochem. Cell Biol.* **43**, 465–469 (2011).

2. U. Knippschild, M. Kruger, J. Richter, P. Xu, B. Garcia-Reyes, C. Peifer, J. Halekotte, V. Bakulev, J. Bischof, The CK1 family: Contribution to cellular stress response and its role in carcinogenesis. *Front. Oncol.* **4**, 1–32 (2014).

3. H. Flotow, P. R. Graves, A. Wang, C. J. Fiol, R. W. Roeske, P. J. Roach, Phosphate groups as substrate determinants for casein kinase I action. *J. Biol. Chem.* **265**, 14264–14269 (1990).

4. A. Rivers, K. F. Gietzen, E. Vielhaber, D. M. Virshup, Regulation of casein kinase I epsilon and casein kinase I delta by an in vivo futile phosphorylation cycle. *J. Biol. Chem.* **273**, 15980–15984 (1998).

5. X. Zeng, K. Tamai, B. Doble, S. Li, H. Huang, R. Habas, H. Okamura, J. Woodgett, X. He, A dual-kinase mechanism for Wnt co-receptor phosphorylation and activation. *Nature* **438**, 873–877 (2005).

6. D. Bhandari, J. Zhang, S. Menon, C. Lord, S. Chen, J. R. Helm, K. Thorsen, K. D. Corbett, J. C. Hay, S. Ferro-Novick, Sit4p/PP6 regulates ER-to-Golgi traffic by controlling the dephosphorylation of COPII coat subunits. *Mol. Biol. Cell* **24**, 2727–2738 (2013).

7. T. Ishiguro, K. Tanaka, T. Sakuno, Y. Watanabe, Shugoshin–PP2A counteracts casein-kinase-1-dependent cleavage of Rec8 by separase. *Nat. Cell Biol.* **12**, 500–506 (2010).

8. R. Narasimamurthy, S. R. Hunt, Y. Lu, J.-M. Fustin, H. Okamura, C. L. Partch, D. B. Forger, J. K. Kim, D. M. Virshup, CK1δ/ε protein kinase primes the PER2 circadian phosphoswitch. *Proc. Natl. Acad. Sci. U.S.A.* **115**, 5986–5991 (2018).

9. W. Swiatek, H. Kang, B. A. Garcia, J. Shabanowitz, G. S. Coombs, D. F. Hunt, D. M. Virshup, Negative regulation of LRP6 function by casein kinase I ϵ phosphorylation. *J. Biol. Chem.* **281**, 12233–12241 (2006).

10. A. E. Johnson, J.-S. Chen, K. L. Gould, CK1 is required for a mitotic checkpoint that delays cytokinesis. *Curr. Biol.* **23**, 1920–1926 (2013).

11. N. Phadnis, L. Cipak, S. Polakova, R. W. Hyppa, I. Cipakova, D. Anrather, L. Karvaiova, K. Mechtler, G. R. Smith, J. Gregan, Casein kinase 1 and phosphorylation of cohesin subunit Rec11 (SA3) promote meiotic recombination through linear element formation. *PLOS Genet.* **11**, e1005225 (2015).

12. T. Sakuno, Y. Watanabe, Phosphorylation of cohesin Rec11/SA3 by casein kinase 1 promotes homologous recombination by assembling the meiotic chromosome axis. *Dev. Cell* **32**, 220–230 (2015).

13. Y. E. Greer, J. S. Rubin, Casein kinase 1 delta functions at the centrosome to mediate Wnt-3a–dependent neurite outgrowth. *J. Cell Biol.* **192**, 993–1004 (2011).

14. Z. C. Elmore, R. X. Guillen, K. L. Gould, The kinase domain of CK1 enzymes contains the localization cue essential for compartmentalized signaling at the spindle pole. *Mol. Biol. Cell* **29**, 1664–1674 (2018).

15. Q. Ye, S. N. Ur, T. Y. Su, K. D. Corbett, Structure of the Saccharomyces cerevisiae Hrr25:Mam1 monopolin subcomplex reveals a novel kinase regulator. *EMBO J.* **35**, 2139–2151 (2016).

16. S. N. Cullati, A. Chaikuad, J.-S. Chen, J. Gebel, L. Tesmer, R. Zhubi, J. Navarrete-Perea, R. X. Guillen, S. P. Gygi, G. Hummer, V. Dötsch, S. Knapp, K. L. Gould, Kinase domain autophosphorylation rewires the activity and substrate specificity of CK1 enzymes. *Mol. Cell* **82**, 2006–2020.e8 (2022).

17. A. Cegielska, K. F. Gietzen, A. Rivers, D. M. Virshup, Autoinhibition of casein kinase I epsilon (CKI epsilon) is relieved by protein phosphatases and limited proteolysis. *J. Biol. Chem.* **273**, 1357–1364 (1998).

18. K. F. Gietzen, D. M. Virshup, Identification of inhibitory autophosphorylation sites in casein kinase I ε. *J. Biol. Chem.* **274**, 32063–32070 (1999).

19. P. R. Graves, P. J. Roach, Role of COOH-terminal phosphorylation in the regulation of casein kinase Iδ. *J. Biol. Chem.* **270**, 21689–21694 (1995).

20. M. F. Hoekstra, N. Dhillon, G. Carmel, A. J. DeMaggio, R. A. Lindberg, T. Hunter, J. Kuret, Budding and fission yeast casein kinase I isoforms have dual-specificity protein kinase activity. *Mol. Biol. Cell* **5**, 877–886 (1994).

21. N. Dhillon, M. F. Hoekstra, Characterization of two protein kinases from Schizosaccharomyces pombe involved in the regulation of DNA repair. *EMBO J.* **13**, 2777–2788 (1994).

22. A. Carpy, K. Krug, S. Graf, A. Koch, S. Popic, S. Hauf, B. Macek, Absolute proteome and phosphoproteome dynamics during the cell cycle of Schizosaccharomyces pombe (fission yeast). *Mol. Cell. Proteomics* **13**, 1925–1936 (2014).

23. A. N. Kettenbach, L. Deng, Y. Wu, S. Baldissard, M. E. Adamo, S. A. Gerber, J. B. Moseley, Quantitative phosphoproteomics reveals pathways for coordination of cell growth and division by the conserved fission yeast kinase Pom1. *Mol. Cell. Proteomics* **14**, 1275–1287 (2015).

24. A. Koch, K. Krug, S. Pengelley, B. Macek, S. Hauf, Mitotic substrates of the kinase aurora with roles in chromatin regulation identified through quantitative phosphoproteomics of fission yeast. *Sci. Signal.* **4**, rs6 (2011).

25. M. P. Swaffer, A. W. Jones, H. R. Flynn, A. P. Snijders, P. Nurse, Quantitative phosphoproteomics reveals the signaling dynamics of cell-cycle kinases in the fission yeast Schizosaccharomyces pombe. *Cell Rep.* **24**, 503–514 (2018).

26. K. Akizuki, T. Toyama, M. Yamashita, Y. Sugiyama, A. Ishida, I. Kameshita, N. Sueyoshi, Facile preparation of highly active casein kinase 1 using Escherichia coli constitutively expressing lambda phosphatase. *Anal. Biochem.* **549**, 99–106 (2018).

27. M. Lambert, J. Gebel, C. Trejtnar, N. Wesch, S. Bozkurt, M. Adrian-Allgood, F. Löhr, C. Münch, V. Dötsch, Fuzzy interactions between the auto-phosphorylated C-terminus and the kinase domain of CK1δ inhibits activation of TAp63α. *Sci. Rep.* **13**, 16423 (2023).

28. J. Gebel, M. Tuppi, A. Chaikuad, K. Hötte, M. Schröder, L. Schulz, F. Löhr, N. Gutfreund, F. Finke, E. Henrich, J. Mezhyrova, R. Lehnert, F. Pampaloni, G. Hummer, E. H. K. Stelzer, S. Knapp, V.

Dötsch, p63 uses a switch-like mechanism to set the threshold for induction of apoptosis. *Nat. Chem. Biol.* **16**, 1078–1086 (2020).

29. J. M. Philpott, A. M. Freeberg, J. Park, K. Lee, C. G. Ricci, S. R. Hunt, R. Narasimamurthy, D. H. Segal, R. Robles, Y. Cai, S. Tripathi, J. A. McCammon, D. M. Virshup, J. C. Chiu, C. Lee, C. L. Partch, PERIOD phosphorylation leads to feedback inhibition of CK1 activity to control circadian period. *Mol. Cell* **83**, 1677–1692.e8 (2023).

30. Y. Shinohara, Y. M. Koyama, M. Ukai-Tadenuma, T. Hirokawa, M. Kikuchi, R. G. Yamada, H. Ukai, H. Fujishima, T. Umehara, K. Tainaka, H. R. Ueda, Temperature-sensitive substrate and product binding underlie temperature-compensated phosphorylation in the clock. *Mol. Cell* **67**, 783–798.e20 (2017).

31. Z. Su, J. Song, Z. Wang, L. Zhou, Y. Xia, S. Yu, Q. Sun, S.-S. Liu, L. Zhao, S. Li, L. Wei, D. A. Carson, D. Lu, Tumor promoter TPA activates Wnt/β-catenin signaling in a casein kinase 1-dependent manner. *Proc. Natl. Acad. Sci. U.S.A.* **115**, E7522–E7531 (2018).

32. G. Wu, H. Huang, J. G. Abreu, X. He, Inhibition of GSK3 phosphorylation of β-catenin via phosphorylated PPPSPXS motifs of Wnt coreceptor LRP6. *PLOS ONE* **4**, e4926 (2009).

33. J.-M. Fustin, R. Kojima, K. Itoh, H.-Y. Chang, S. Ye, B. Zhuang, A. Oji, S. Gibo, R. Narasimamurthy, D. Virshup, G. Kurosawa, M. Doi, I. Manabe, Y. Ishihama, M. Ikawa, H. Okamura, Two Ck1δ transcripts regulated by m6A methylation code for two antagonistic kinases in the control of the circadian clock. *Proc. Natl. Acad. Sci. U.S.A.* **115**, 5980–5985 (2018).

34. Y. Isojima, M. Nakajima, H. Ukai, H. Fujishima, R. G. Yamada, K. Masumoto, R. Kiuchi, M. Ishida, M. Ukai-Tadenuma, Y. Minami, R. Kito, K. Nakao, W. Kishimoto, S.-H. Yoo, K. Shimomura, T. Takao, A. Takano, T. Kojima, K. Nagai, Y. Sakaki, J. S. Takahashi, H. R. Ueda, CKIε/δ-dependent phosphorylation is a temperature-insensitive, period-determining process in the mammalian circadian clock. *Proc. Natl. Acad. Sci. U.S.A.* **106**, 15744–15749 (2009).

35. J. M. Philpott, R. Narasimamurthy, C. G. Ricci, A. M. Freeberg, S. R. Hunt, L. E. Yee, R. S. Pelofsky, S. Tripathi, D. M. Virshup, C. L. Partch, Casein kinase 1 dynamics underlie substrate selectivity and the PER2 circadian phosphoswitch. *eLife* **9**, e52343 (2020).

36. J. L. Johnson, T. M. Yaron, E. M. Huntsman, A. Kerelsky, J. Song, A. Regev, T.-Y. Lin, K. Liberatore, D. M. Cizin, B. M. Cohen, N. Vasan, Y. Ma, K. Krismer, J. T. Robles, B. van de Kooij, A. E. van Vlimmeren, N. Andrée-Busch, N. F. Käufer, M. V. Dorovkov, A. G. Ryazanov, Y. Takagi, E. R. Kastenhuber, M. D. Goncalves, B. D. Hopkins, O. Elemento, D. J. Taatjes, A. Maucuer, A. Yamashita, A. Degterev, M. Uduman, J. Lu, S. D. Landry, B. Zhang, I. Cossentino, R. Linding, J. Blenis, P. V. Hornbeck, B. E. Turk, M. B. Yaffe, L. C. Cantley, An atlas of substrate specificities for the human serine/threonine kinome. *Nature* **613**, 759–766 (2023).

37. T. M. Yaron, B. E. Heaton, T. M. Levy, J. L. Johnson, T. X. Jordan, B. M. Cohen, A. Kerelsky, T.-Y. Lin, K. M. Liberatore, D. K. Bulaon, S. J. Van Nest, N. Koundouros, E. R. Kastenhuber, M. N. Mercadante, K. Shobana-Ganesh, L. He, R. E. Schwartz, S. Chen, H. Weinstein, O. Elemento, E. Piskounova, B. E. Nilsson-Payant, G. Lee, J. D. Trimarco, K. N. Burke, C. E. Hamele, R. R. Chaparian, A. T. Harding, A. Tata, X. Zhu, P. R. Tata, C. M. Smith, A. P. Possemato, S. L. Tkachev, P. V. Hornbeck, S. A. Beausoleil, S. K. Anand, F. Aguet, G. Getz, A. D. Davidson, K. Heesom, M. Kavanagh-Williamson, D. A. Matthews, B. R. TenOever, L. C. Cantley, J. Blenis, N. S. Heaton, Host protein kinases required for SARS-CoV-2 nucleocapsid phosphorylation and viral replication. *Sci. Signal.* **15**, eabm0808 (2022).

38. C.-M. Cruciat, C. Dolde, R. E. A. de Groot, B. Ohkawara, C. Reinhard, H. C. Korswagen, C. Niehrs, RNA helicase DDX3 is a regulatory subunit of casein kinase 1 in Wnt–β-catenin signaling. *Science* **339**, 1436–1441 (2013).

39. R. Harold, N. K. Tulsian, R. Narasimamurthy, N. Yaitanes, M. G. A. Hernandez, H.-W. Lee, D. M. Virshup, C. L. Partch, Isoform-specific C-terminal phosphorylation drives autoinhibition of casein kinase 1. bioRxiv 2023.04.24.538174 [Preprint] (2023). https://doi.org/10.1101/2023.04.24.538174.

40. A. Cegielska, D. M. Virshup, Control of simian virus 40 DNA replication by the HeLa cell nuclear kinase casein kinase I. *Mol. Cell. Biol.* **13**, 1202–1211 (1993).

41. H. Flotow, P. J. Roach, Role of acidic residues as substrate determinants for casein kinase I. *J. Biol. Chem.* **266**, 3724–3727 (1991).

42. F. Liu, D. M. Virshup, A. C. Nairn, P. Greengard, Mechanism of regulation of casein kinase I activity by group I metabotropic glutamate receptors. *J. Biol. Chem.* **277**, 45393–45399 (2002).

43. C. L. Partch, K. F. Shields, C. L. Thompson, C. P. Selby, A. Sancar, Posttranslational regulation of the mammalian circadian clock by cryptochrome and protein phosphatase 5. *Proc. Natl. Acad. Sci. U.S.A.* **103**, 10467–10472 (2006).

44. J. Bischof, S.-J. Randoll, U. Knippschild, CK1δ kinase activity is modulated by Chk1-mediated phosphorylation. *PLOS ONE* **8**, e68803 (2013).

45. G. Giamas, H. Hirner, L. Shoshiashvili, A. Grothey, S. Gessert, M. Kühl, D. Henne-Bruns, C. E. Vorgias, U. Knippschild, Phosphorylation of CK1δ: identification of Ser370 as the major phosphorylation site targeted by PKA in vitro and in vivo. *Biochem. J.* **406**, 389–398 (2007).

46. C. Ianes, P. Xu, N. Werz, Z. Meng, D. Henne-Bruns, J. Bischof, U. Knippschild, CK1δ activity is modulated by CDK2/E- and CDK5/p35-mediated phosphorylation. *Amino Acids* **48**, 579–592 (2016).

47. Z. Meng, J. Bischof, C. Ianes, D. Henne-Bruns, P. Xu, U. Knippschild, CK1δ kinase activity is modulated by protein kinase C α (PKCα)-mediated site-specific phosphorylation. *Amino Acids* **48**, 1185–1197 (2016).

48. T. D. Pollard, A guide to simple and informative binding assays. *Mol. Biol. Cell* **21**, 4061–4067 (2010).

49. S. Moreno, A. Klar, P. Nurse, Molecular genetic analysis of fission yeast Schizosaccharomyces pombe, in *Methods in Enzymology* (Academic Press, 1991), vol. 194 of *Guide to Yeast Genetics and Molecular Biology*, pp. 795–823.

50. J. B. Keeney, J. D. Boeke, Efficient targeted integration at leu1-32 and ura4-294 in Schizosaccharomyces pombe. *Genetics* **136**, 849–856 (1994).

51. J. Bähler, J.-Q. Wu, M. S. Longtine, N. G. Shah, A. Mckenzie III, A. B. Steever, A. Wach, P. Philippsen, J. R. Pringle, Heterologous modules for efficient and versatile PCR-based gene targeting in Schizosaccharomyces pombe. *Yeast* **14**, 943–951 (1998).

52. M. A. Harris, K. M. Rutherford, J. Hayles, A. Lock, J. Bähler, S. G. Oliver, J. Mata, V. Wood, Fission stories: using PomBase to understand Schizosaccharomyces pombe biology. *Genetics* **220**, iyab222 (2022).

53. K. I. Gould, S. Moreno, D. J. Owen, S. Sazer, P. Nurse, Phosphorylation at Thr167 is required for Schizosaccharomyces pombe p34cdc2 function. *EMBO J.* **10**, 3297–3309 (1991).

54. S. E. Plyte, A. Feoktistova, J. D. Burke, J. R. Woodgett, K. L. Gould, Schizosaccharomyces pombe skp1+ encodes a protein kinase related to mammalian glycogen synthase kinase 3 and complements a cdc14 cytokinesis mutant. *Mol. Cell. Biol.* **16**, 179–191 (1996).

55. J.-S. Chen, M. R. Broadus, J. R. McLean, A. Feoktistova, L. Ren, K. L. Gould, Comprehensive proteomics analysis reveals new substrates and regulators of the fission yeast Clp1/Cdc14 phosphatase. *Mol. Cell. Proteomics* **12**, 1074–1086 (2013).

56. W. J. Boyle, P. van der Geer, T. Hunter, Phosphopeptide mapping and phosphoamino acid analysis by two-dimensional separation on thin-layer cellulose plates, in *Methods in Enzymology* (Academic Press, 1991), vol. 201 of *Protein Phosphorylation Part B: Analysis of Protein Phosphorylation, Protein Kinase Inhibitors, and Protein Phosphatases*, pp. 110–149.

57. L. A. Kelley, S. Mezulis, C. M. Yates, M. N. Wass, M. J. E. Sternberg, The Phyre2 web portal for protein modeling, prediction and analysis. *Nat. Protoc.* **10**, 845–858 (2015).

58. A. M. Long, H. Zhao, X. Huang, Structural basis for the potent and selective inhibition of casein kinase 1 epsilon. *J. Med. Chem.* **55**, 10307–10311 (2012).

59. A. Bimbo, Y. Jia, S. L. Poh, R. K. M. Karuturi, N. den Elzen, X. Peng, L. Zheng, M. O’Connell, E. T. Liu, M. K. Balasubramanian, J. Liu, Systematic deletion analysis of fission yeast protein kinases. *Eukaryot. Cell* **4**, 799–813 (2005).
